# Construction of Competitive Endogenous RNA Network and Verification of 3-Key LncRNA Signature Associated With Distant Metastasis and Poor Prognosis in Patients With Clear Cell Renal Cell Carcinoma

**DOI:** 10.3389/fonc.2021.640150

**Published:** 2021-03-24

**Authors:** Yang Su, Tianxiang Zhang, Jieqiong Tang, Li Zhang, Song Fan, Jun Zhou, Chaozhao Liang

**Affiliations:** ^1^Department of Urology, The First Affiliated Hospital of Anhui Medical University, Hefei, China; ^2^Anhui Province Key Laboratory of Genitourinary Diseases, Anhui Medical University, Hefei, China; ^3^The Institute of Urology, Anhui Medical University, Hefei, China; ^4^The Second Clinical Medical College, Anhui Medical University, Hefei, China

**Keywords:** distant metastasis, prognosis, clear cell renal cell carcinoma, lncRNA, competitive endogenous RNA regulatory network

## Abstract

Clear cell renal cell carcinoma (ccRCC) is a common malignancy with high distant metastasis rate. Long non-coding RNAs (LncRNAs) are reported to be upregulated or downregulated in multiple cancers and play a crucial role in the metastasis of tumors or prognosis. Therefore, the purpose of our study is to construct a prognostic signature for ccRCC based on distant metastasis-related lncRNAs and explore the involved potential competitive endogenous RNA (ceRNA) network. The differentially expressed genes (DEGs) screened from the database of the cancer genome atlas (TCGA) were used to construct a co-expression network and identify the distant metastasis-related module by weighted gene co-expression network analysis (WGCNA). Key genes with metastatic and prognostic significance were identified through rigorous screening, including survival analysis, correlation analysis, and expression analyses in stage, grade, and distant metastasis, and were verified in the data set of gene expression omnibus (GEO) and the database from gene expression profiling interactive analysis (GEPIA). The potential upstream miRNAs and lncRNAs were predicted *via* five online databases and LncBase. Here, we constructed a ceRNA network of key genes that are significantly associated with the distant metastasis and prognosis of patients with ccRCC. The distant metastasis-related lncRNAs were used to construct a risk score model through the univariate, least absolute shrinkage selection operator (LASSO), and multivariate Cox regression analyses, and the patients were divided into high- and low-risk groups according to the median of the risk score. The Kaplan–Meier survival analysis demonstrated that mortality was significantly higher in the high-risk group than in the low-risk group. Considering the other clinical phenotype, the Cox regression analyses indicated that the lncRNAs model could function as an independent prognostic factor. Quantitative real-time (qRT)-PCR in the tissues and cells of ccRCC verified the high-expression level of three lncRNAs. Gene set enrichment analysis (GSEA) revealed that the lncRNA prognostic signature was mainly enriched in autophagy- and immune-related pathways, indicating that the autophagy and immune functions may play an important role in the distant metastasis of ccRCC. In summary, the constructed distant metastasis-related lncRNA signature could independently predict prognosis in patients with ccRCC, and the related ceRNA network provided a new sight on the potential mechanism of distant metastasis and a promising therapeutic target for ccRCC.

## Introduction

Renal cell carcinoma (RCC), the third most common malignancy of the urinary system, also belongs to the ninth most common cancer, worldwide (1). It is estimated that RCC accounts for 73,750 new cases and 14,830 death cases in the United States in 2020 (2). Clear cell renal cell carcinoma (ccRCC), as the most common pathological type of RCC, has more malignant features and a worse prognosis compared to other types of RCC, accounting for the majority of RCC-related deaths (3). Therapeutically, the ccRCC is insensitive to radiotherapy and chemotherapy. The nephrectomy (radical or partial) and ablation techniques may be the effective treatment for localized RCC; however, recurrence or metastasis still occurs in about 20–40% of patients after surgery (4). Patients with ccRCC and with distant metastasis almost lose the indication of surgery, and mainly rely on conservative treatment; their 5-year survival rate is <10% despite the use of one or more combination therapies, including cytoreductive nephrectomy or metastasectomy, VEGFR inhibitors, mTOR inhibitors, and immunotherapy (5). Therefore, in order to improve the prognosis of patients with ccRCC and distant metastasis, it is very urgent to explore the mechanism of the development of ccRCC and identify the potential therapy target associated with tumorigenesis, distant metastasis, and prognosis.

Long non-coding RNAs (lncRNAs) are a class of non-coding RNA transcripts that have more than 200 nucleotides in length (6). Currently, lncRNAs are considered to play an important role in the biological behavior of cancers and have been extensively studied in the expression profiles of various cancers (7). MicroRNAs (miRNAs), a range of small endogenous non-coding RNAs of ~22 nucleotides in length, have emerged as one of the most important players in the initiation and progression of cancers in recent decades (8). Salmena et al. (9) proposed the hypothesis of competing endogenous RNA (ceRNA), which suggests that miRNAs and lncRNAs as well as other non-coding RNAs are able to cross-talk by competitively binding to shared miRNAs through microRNA response elements (MREs), thereby exerting their biological effects. Nowadays, increasing studies have proven that the ceRNA regulation theory plays a critical role in the development of cancers (10). Furthermore, weighted gene co-expression network analysis (WGCNA), a newly invented hierarchical clustering method, focused on the whole genome information to summarize the signature of gene networks, which can avoid bias and subject judgment, has been used to describe the connectivity of gene clusters inside the comprehensive network and assess the correlations of gene modules with different clinical features (11).

Although previous studies have identified that certain lncRNAs could be used as biomarkers to predict the progression or prognosis in patients with ccRCC (12, 13), the distant metastasis-related lncRNA signature and its potential regulatory network have not been fully explored. Considering this situation, we accurately constructed a ceRNA network that was closely associated with distant metastasis and prognosis of patients with ccRCC *via* a series of strict screening and verification. Furthermore, a distant metastasis-related lncRNA signature that can independently predict the poor prognosis of patients with ccRCC was extracted from the ceRNA network and validated in the tissues and cell lines of ccRCC. The prognostic model will help to improve the clinical assessment of patients with advanced ccRCC. In addition, we explored the potential pathways of the lncRNA signature and found that the prognostic signature might promote distant metastasis of ccRCC by regulating autophagy and immunity.

## Materials and Methods

### Data Collection and Pre-processing

**Figure 1:**
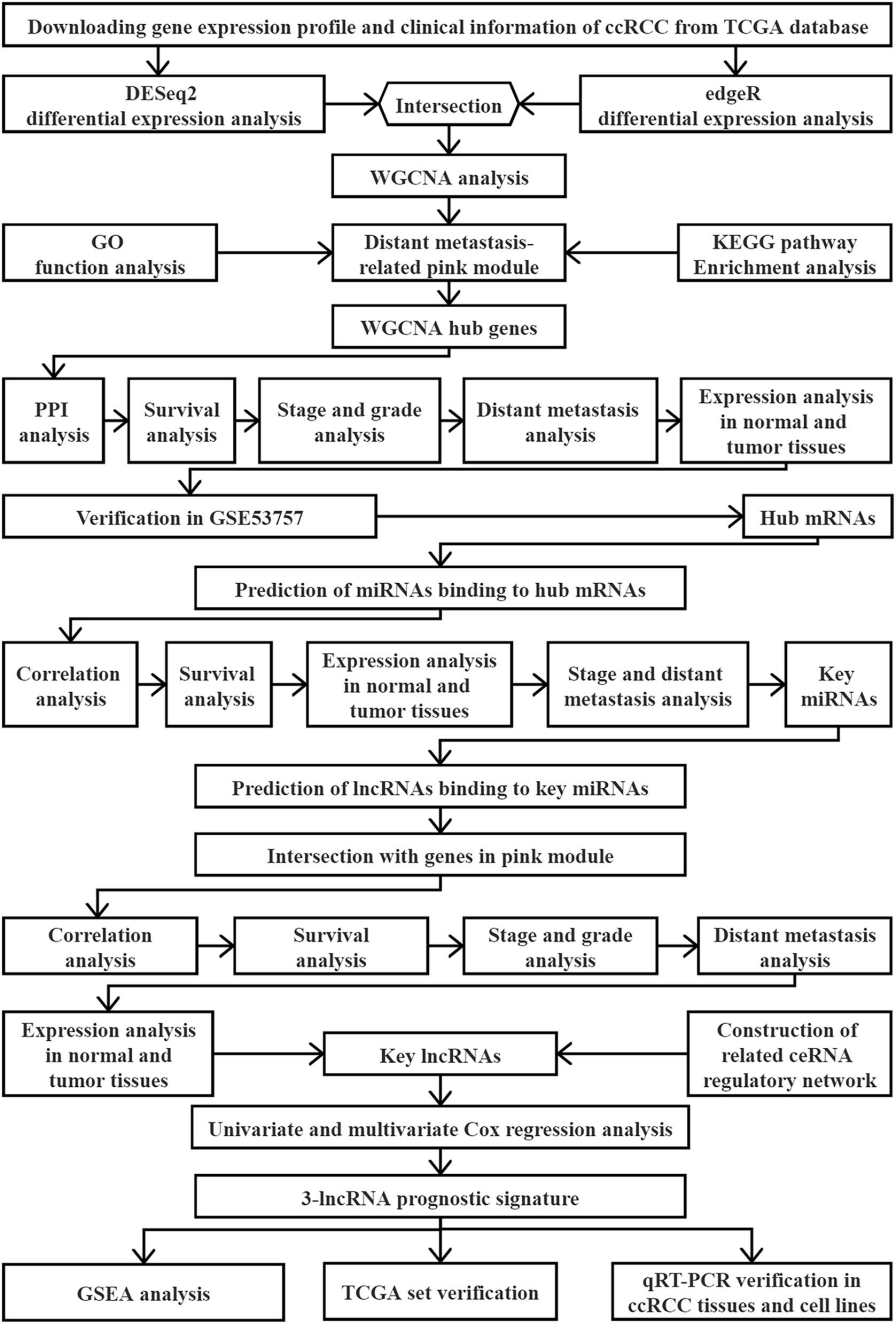
Flow chart of the study.

A flow chart of the study design is shown in Figure 1. The data of 539 human ccRCC samples and 72 normal samples downloaded from the database of the cancer genome atlas (TCGA) (https://portal.gdc.cancer.gov), including the data of the RNA-sequencing (Seq) and corresponding clinical information (Table 1), was used to screen the differentially expressed genes (DEGs). Meanwhile, the microarray data set GSE53757 of 72 paired ccRCC/normal tissue samples was downloaded from the database of the gene expression omnibus (GEO) (http://www.ncbi.nlm.nih.gov/geo/) in Affymetrix Human Genome U133 Plus 2.0 Array platform, providing validation for screened hub genes. All the raw expression data were normalized with R package “limma” (14) and the value of gene expression in the data set from TCGA was transformed as log_2_ (x + 1) counts.

**Table 1:** Summary of the clinical characteristics in ccRCC patients.

**Characteristics**	**Case**	**Percentage (%)**
**Age (Years)**		
≤40	26	4.84
>40	511	95.16
**Gender**		
Female	191	35.57
Male	346	64.43
**Race**		
Asian	8	1.49
Black	56	10.43
White	466	86.78
Unknown	7	1.30
**Pathological stage**		
Stage I	269	50.09
Stage II	57	10.61
Stage III	125	23.28
Stage IV	83	15.46
Unknown	3	0.056
**Histologic grade**		
G1	14	2.61
G2	230	42.83
G3	207	38.55
G4	78	14.52
Unknown	8	1.49
**Tumor status**		
T1	275	51.21
T2	69	12.85
T3	182	33.89
T4	11	2.05
**Lymph node status**		
N0	240	44.69
N1	17	3.17
Unknown	280	52.14
**Distant metastasis**		
M0	426	79.33
M1	79	14.71
Unknown	32	5.96

### Identification of DEGs

R packages, such as “edgeR” (15) and “DESeq2” (16) were used to separately screen DEGs between 539 ccRCC tissues and 72 normal tissues in the TCGA data set. The threshold was set to |logFC| > 1 and the adjusted *p* < 0.05. A web tool for creating Venn diagrams (http://bioinformatics.psb.ugent.be/webtools/Venn/) was used to find the intersection of DEGs that commonly upregulated or downregulated in the two R packages mentioned above. Volcano plots were generated based on the “Enhanced Volcano” package, and a heatmap was generated using the “heatmap” in the R package.

### Establishment of WGCNA

The weighted gene co-expression network analysis (WGCNA) of DEGs was constructed by “WGCNA,” an R package (17). First, a sample tree was constructed using the function, “goodSamplesGenes” in the “WGCNA” package to check all samples for outliers. The outlier in the sample tree was then removed based on the cut height. Subsequently, a correlation matrix was constructed by calculating Pearson's correlations of the processed genes. After iteration with a set of soft-thresholding powers (range 1–20), an appropriate power of β was chosen as the soft-thresholding parameter to construct an adjacency matrix. Next, the adjacency matrix was transformed into a topological overlap matrix (TOM) that was close to a scale-free network. In the co-expression network, genes with highly absolute correlations were clustered into the same module to generate a cluster dendrogram. The parameters are described as below:

TOMType = unsigned, minModuleSize = 35, deepSplit = 2, mergeCutHeight = 0.15.

### Identify Significant Relevant Module

The relationships between co-expression modules and clinical traits were calculated by Pearson's correlation coefficient and plotted using a heatmap. In the current study, distant metastasis was selected as the interested clinical phenotype for subsequent analysis. Furthermore, in the linear regression between gene expression and clinical phenotype, gene significance (GS) was calculated as the absolute value of the correlation between the gene expression profile and each external trait. Module membership (MM) represents the correlation between a single gene and the module in which it is located, indicating whether it is consistent with the trend of the module.

### Functional Enrichment Analysis

To investigate the biological function of the pink module that is significantly associated with the distant metastasis of ccRCC, the R package, “clusterProfiler” (18) was used for gene ontology (GO) function analysis and Kyoto Encyclopedia of Genes and Genomes (KEGG) pathway enrichment analysis. The “enrichplot” package was used for the visualization of KEGG function analysis, and the “GOplot” package was used to generate chord diagrams of GO function analysis. The cut-off criterion denoted that the *p* < 0.05.

### Hub mRNAs Screened by WGCNA and Protein–Protein Interaction

Hub genes, highly interconnected with other genes in a module, are considered to be functionally significant. In the current study, genes in the key module were imported into Cytoscape 3.7.1 (19) to construct the WGCNA network. The degree of nodes in the network was calculated by “NetworkAnalyzer” in the Cytoscape. According to the degree, genes ranked top 54 (degree > 200) in the WGCNA network were chosen as the candidates for further analysis. Subsequently, the list of 54 hub genes was uploaded to the STRING database (https://string-db.org/) to obtain the relationship between mRNAs, thereby constructing a protein–protein interaction (PPI) network (20). An interaction score of 0.99 was set as the threshold for the hub mRNAs in the PPI network, and the Cytoscape 3.7.1 was used to visualize the network diagram for PPI analysis.

### Verify the Expression Patterns and Prognostic Value of Hub mRNAs

To validate the expression level of hub mRNAs in different stages or in the normal and ccRCC samples, the GSE53757 was chosen as the external validation data set. Subsequently, we used the database from gene expression profiling interactive analysis (GEPIA) (http://gepia.cancer-pku.cn/) to verify the prognostic value of hub genes in the overall survival (OS) and disease-free survival (DFS).

### Identification of Key miRNAs by Database Prediction, Correlation Analysis, Survival Analysis, and Expression Analysis

In this study, a total of 5 databases, containing miRTarBase (http://miRTarBase.mbc.nctu.edu.tw/.), TargetScan (http://www.targetscan.org/), miRWalk (http://mirwalk.umm.uni-heidelberg.de/), starBase (http://starbase.sysu.edu.cn/), and microT (http://www.microrna.gr/webServer) were employed to predict the upstream miRNAs potentially binding to hub mRNAs. Only those miRNAs that appeared in more than one predicting database were selected for further analysis.

The starBase (21) database is an open-source platform for identifying the RNA–RNA interaction networks and providing platforms to perform the survival and differential expression analysis of lncRNAs, miRNAs, and mRNAs. Therefore, we also used the starBase to analyze the expression correlation between miRNAs and mRNAs, and *R* < −0.1 and *p* < 0.05 were set as the cut-off criteria. The starBase database was also introduced to conduct survival analysis and expression analysis between ccRCC tissues and adjacent tissues for predicted miRNAs. Additionally, the expression information of targeted miRNAs in different stages, distant metastasis tissues, and non-distant metastasis tissues were downloaded from the LinkedOmics database (http://www.linkedomics.org).

### Identification of Distant Metastasis-Related lncRNAs

The LncBase (www.microrna.gr/LncBase) was introduced to predict the upstream lncRNAs potentially targeting the key miRNAs. The expression correlation between lncRNAs and miRNAs was analyzed using the starBase database. The GEPIA database was utilized to evaluate the prognostic value of upstream lncRNAs in OS and DFS. Furthermore, the expression analysis of lncRNAs in different stages, different grades, ccRCC samples and adjacent samples, distant metastasis tissues, and in non-distant metastasis tissues was conducted in the database from TCGA.

### Construction of the 3-lncRNA Prognostic Signature

The prognostic significance of identified three distant metastasis-related lncRNAs was firstly validated by univariate Cox regression analysis. Then, least absolute shrinkage and selection operator (LASSO) regression analysis was performed to enhance the prediction accuracy of the prognostic model using the “glmnet” (22) R package. Subsequently, a risk score model was constructed by multivariate Cox regression analysis. Based on the expression level of each lncRNA and the regression coefficient obtained from multivariate Cox regression analysis, the risk score was calculated as follows:

$$\begin{array}{l} \text{Riskscore}=\sum_{\text{i}=1}^{\text{n}}\text{Coe}{\text{f}}_{\text{i}}\times \text{Ex}{\text{p}}_{\text{i}} \end{array}$$

where, Coef is the regression coefficient of each lncRNA, and Exp represents the expression level of each lncRNA. Patients were divided into high- and low-risk groups according to the median of risk score, and Kaplan–Meier survival curve was used to assess the survival differences between high- and low-risk groups using the R package, “survminer.” Furthermore, the predictive power of the lncRNA signature was assessed by calculating the area under the curve (AUC) of 3 years dependent receiver operating characteristic (ROC) curve using the “survivalROC” package. In addition, the clinical variables that were used to interact with the risk score in univariate and multivariate Cox regression analyses include age, gender, race, grade, stage, N stage, and M stage.

### Gene Set Enrichment Analysis

To identify the significant gene sets enriched in high- and low-risk groups, GSEA (http://software.broadinstitute.org/gsea/index.jsp) was conducted in the data set from TCGA. The annotated gene set list from c2.cp.kegg.v7.2.symbols.gmt was selected as the reference gene set. A false discovery rate (FDR) < 0.25 and a nominal *p* < 0.05 were considered as the cut-off criteria of enriched gene sets after performing 1,000 permutations. The results of GSEA were visualized using the EnrichmentMap (23) in Cytoscape 3.7.1.

### Collection of Specimens From Clinical Tissue

A total of 30 matched tumor/normal tissue samples were collected from patients with ccRCC in the operating room of the First Affiliated Hospital of Anhui Medical University (Hefei, China). The inclusion criteria of tissue specimens included as follows: (1) pathological diagnosis for ccRCC; (2) except ccRCC with no other malignancy; (3) consistent with radical nephrectomy or partial nephrectomy indications; (4) no radiotherapy and chemotherapy before surgery. All patients signed informed consent forms before surgery, and pathological diagnosis of ccRCC was confirmed by two senior pathologists from our institution, after surgery. The pathological stages of 30 patients with ccRCC were as follows: 19 for Stage I, 8 for Stage II, 2 for Stage III, and 1 for Stage IV. Specimens were immediately frozen in liquid nitrogen as soon as they were removed and stored at −80°C until RNA extraction. The study was approved by the Ethical Committee of Human Research of the hospital.

### Acquisition and Culture of Cell Lines

The human ccRCC cell lines, such as 786-O, A498, ACHN, Caki-1, and the normal renal tubular epithelial cell line, such as HK-2 were acquired from the Cell Bank of Wuhan University (Wuhan, China). Among them, the 786-O, A498, ACHN, and Caki-1 were cultured in RPMI 1640 medium (Gibco Laboratories, MD, USA) with 10% fetal bovine serum (FBS) (ScienCell Research Laboratories, CA, USA) and 1% penicillin-streptomycin (P/S), while the HK-2 cell line was cultured in Dulbecco's Modified Eagle Medium (DMEM) (Gibco Laboratories, MD, USA) with 10% FBS and 1% P/S. All cell lines were cultured at 37°C with 5% CO_2_ and the culture medium was replaced every 3 days.

### Quantitative Real-Time Polymerase Chain Reaction

Total RNA from the tissue samples of ccRCC and cell lines was extracted using Trizol reagent (Invitrogen, Carlsbad, CA, USA). The reverse transcription reactions were performed using a PrimeScriptTM RT reagent kit (Takara, Gunma, Japan) according to the instructions of the manufacturer, and quantitative real-time (qRT)-PCR was prepared at a final volume of 20 μl using an SYBR Green Mix (Takara, Gunma, Japan). The reactions were measured on an AB7500 platform (Thermo Fisher Scientific, MA, USA). The 2^−ΔΔCT^ method was used to determine the relative gene expression level, and Glyceraldehyde 3-phosphate dehydrogenase (GAPDH) was used as an internal control to normalize the data. The primers used for these reactions are listed in Table 2.

**Table 2:** Primers for qRT-PCR of the 3 distant metastasis-related lncRNAs.

**Symbol**	**Forward primer**	**Reverse primer**
LINC01234	5′-GAGGAGTCTCTCGAAATTCAGCA-3′	5′-TGGTCAGATGGGTGGTTGTTT-3′
LINC02577	5′-GTATCTTGTGCCGACCTCCTATC-3′	5′-TCACTCCAACTTCTGCCTTCGT-3′
LINC02609	5′-CAGCGCCCGTTTATTTGAG-3′	5′-AGTGCTCCTGGCTTCTTCTTGTA-3′
GAPDH	5′-GAGAAGGCTGGGGCTCATTT-3′	5′-TAAGCAGTTGGTGGTGCAGG-3′

### Statistical Analysis

Statistical analyses were performed with R software (Version 3.6.1) and GraphPad 7.0. Survival analysis in the data set from TCGA was performed with the R package, “survminer.” One-way ANOVA was used to examine the significance of expression differences among pathological stages or histologic grades. Pearson's correlation test was used to analyze the expression correlation between miRNAs and lncRNAs or mRNAs. Student's *t*-test was used to compare paired data, such as data from ccRCC tissues and adjacent tissues or data from samples of distant metastasis and non-distant metastasis. The value, *p* < 0.05 was considered statistically significant.

## Results

### Screening of DEGs

**Figure 2:**
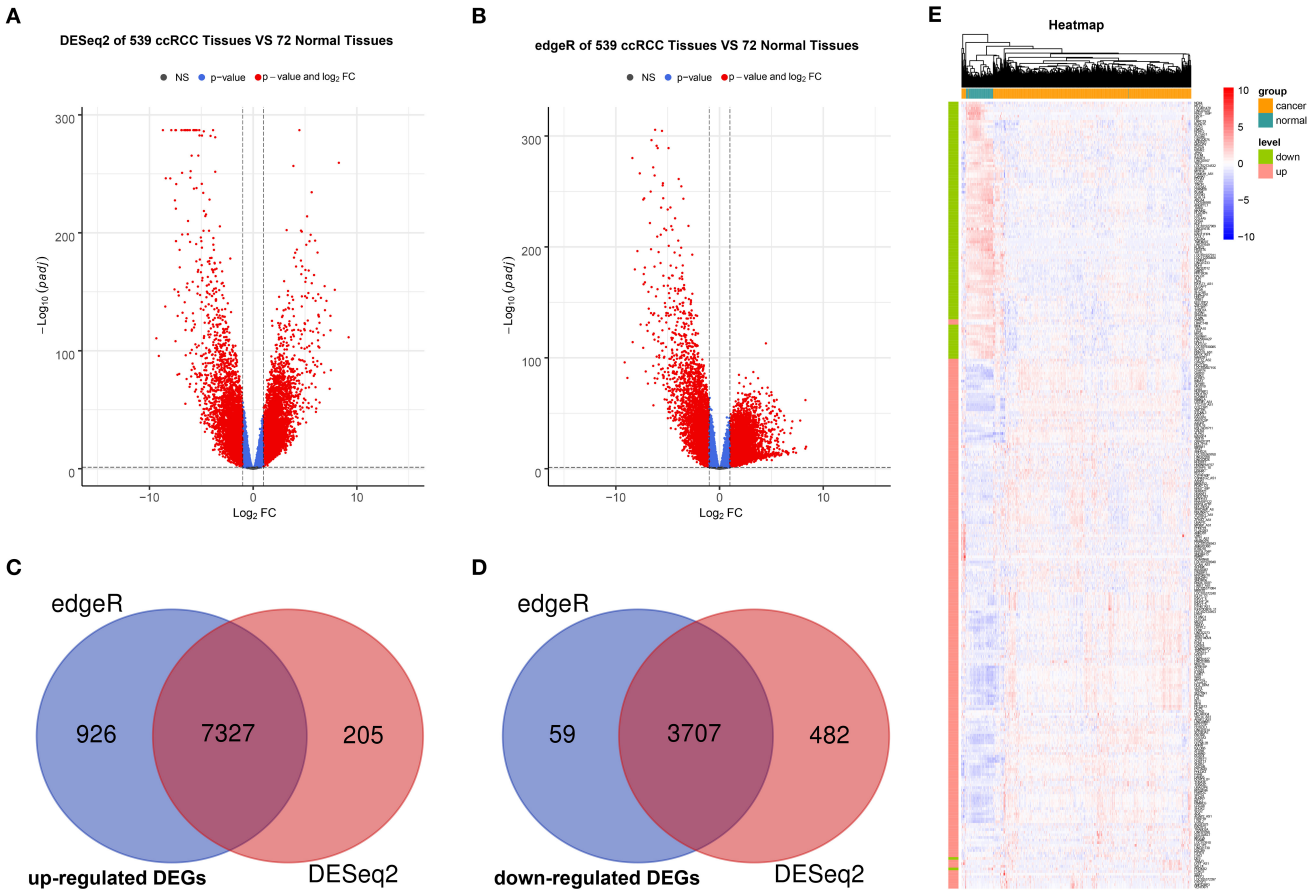
Identification and validation of DEGs. Volcano maps of DEGs screened by “DeSeq2” package **(A)** and “edgeR” package **(B)**. The red nodes represent DEGs. The cutoff values are log|FC| > 1 and the adjusted *p* < 0.05. Venn diagram of common upregulated DEGs **(C)** and common downregulated DEGs **(D)** in 2 R packages. **(E)** Heatmap based on DEGs between 539 ccRCC tissues and 72 normal tissues. Each row represents a screened DEG, in which green bar represents downregulated DEGs and red bar represents upregulated DEGs. Each column represents a tissue sample, where blue bar represents normal tissues and orange bar represents ccRCC tissues. In the heatmap, blue represents negative correlation, while red represents positive correlation. DEGs, differentially expressed genes; ccRCC, clear cell renal cell carcinoma.

The R packages, such as “DEseq2” and “edgeR” were used to screen for DEGs between ccRCC and normal tissues in TCGA, where a total of 11,721 (7,532 upregulated and 4,189 downregulated) and 12,019 (8,253 upregulated and 3,766 downregulated) DEGs were obtained separately. Next, we acquired the upregulated and downregulated DEGs that co-exist in the two analysis methods, where 11,034 DEGs (7,327 upregulated and 3,707 downregulated) were eventually obtained. Volcano plots were utilized to illustrate the distribution of each gene (Figures 2A,B). Venn diagrams were plotted to find the intersection of upregulated and downregulated DEGs separately (Figures 2C,D). Finally, the expression levels of DEGs between ccRCC and normal tissues were displayed using the heatmap in Figure 2E.

### Division of Co-expression Modules

**Figure 3:**
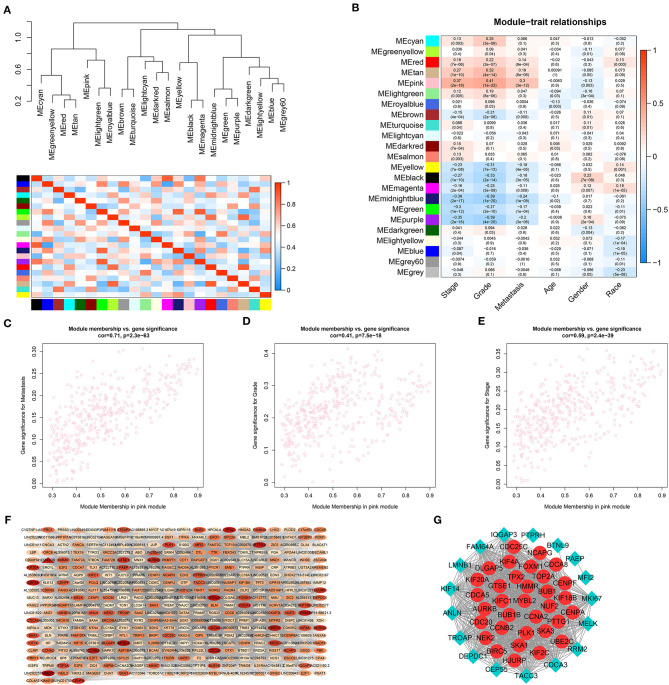
Identification and validation of the distant metastasis-related module and PPI network construction. **(A)** Hierarchical clustering tree and heatmap plot of module eigengene. Each branch of the hierarchical clustering tree represents a module eigengene. Each row or column in the heatmap plot of topological overlap corresponds to one module eigengene labeled by a distinct color. In the heatmap, blue represents negative correlation, while red represents positive correlation. **(B)** Heatmap of module-trait correlation. Each row represents a co-expressed module and each column indicates a clinical trait. Each cell contains Pearson's correlation between a co-expressed module and a clinical feature and the corresponding *p* value is in bracket. Scatterplots of GS for distant metastasis **(C)**, histologic grade **(D)** and pathological stage **(E)** vs. MM in pink module. **(F)** WGCNA co-expression network of pink module. Triangle represents lncRNA and oval stands for other genes. The color of the nodes from white to red indicates the degree of gene connectivity from low to high. **(G)** PPI network of WGCNA hub genes. The red node represents the hub mRNA in the network and the blue diamond stands for non-hub mRNAs in the network. The value of *p* < 0.05 is considered statistically significant. GS, gene significance; MM, module membership; WGCNA, weighted gene correlation network analysis; PPI, protein–protein interaction.

To further identify distant metastasis-related modules, we first performed a hierarchical clustering analysis. Samples with a distance >300 were screened as outliers, and the TCGA-CJ-4642 sample was ultimately excluded (Supplementary Figure 1A). Then, the R package, “WGCNA” was used to construct the WGCNA. In this study, the power of β = 4 (scale-free *R*^2^ = 0.88) was chosen as the soft-thresholding value to ensure a scale-free network (Supplementary Figures 1B,C), and we got 23 co-expression modules by the average linkage hierarchical clustering (Supplementary Figure 1D, Supplementary Table 1). The non-co-expressed genes were grouped into gray module and removed from further analysis. The eigengene adjacency heatmap showed that the expression pattern of each module was independent of each other (Figure 3A). The heatmap of 5,000 randomly selected DEGs showed high degree of topological overlap of co-expression genes in each module (Supplementary Figure 1E).

### Identification of the Key Pink Module

To identify genes associated with distant metastasis of ccRCC, we analyzed the connection between co-expression modules and clinical phenotype using Pearson's correlation. As a result, distant metastasis (*r* = 0.3, *p* = 3e-12) was most significantly associated with pink module. Furthermore, pink module was also most significantly related to histologic grade (*r* = 0.41, *p* = 1e-22) and pathological stage (*r* = 0.37, *p* = 2e-18, Figure 3B). In a module related to a trait of interest, genes with high module membership (MM) tended to have high gene significance (GS), implying that hub genes of the co-expression module tend to be highly correlated with the selected clinical characteristics. The scatterplots of MM vs. GS consistently revealed that MM in the pink module was highly correlated with distant metastasis (cor = 0.71, *p* = 2.3e-63), histologic grade (cor = 0.41, *p* = 7.5e-18) and pathological stage (cor = 0.59, *p* = 2.4e-39, Figures 3C–E), while was not highly correlated with age (cor = 0.14, *p* = 0.0048), gender (cor = −0.012, *p* = 0.81), and race (cor = −0.18, *p* = 0.00027, Supplementary Figures 1F–H). Taking together, we chose the pink module for the next analysis.

### Functional Annotation of Pink Module

**Figure 4:**
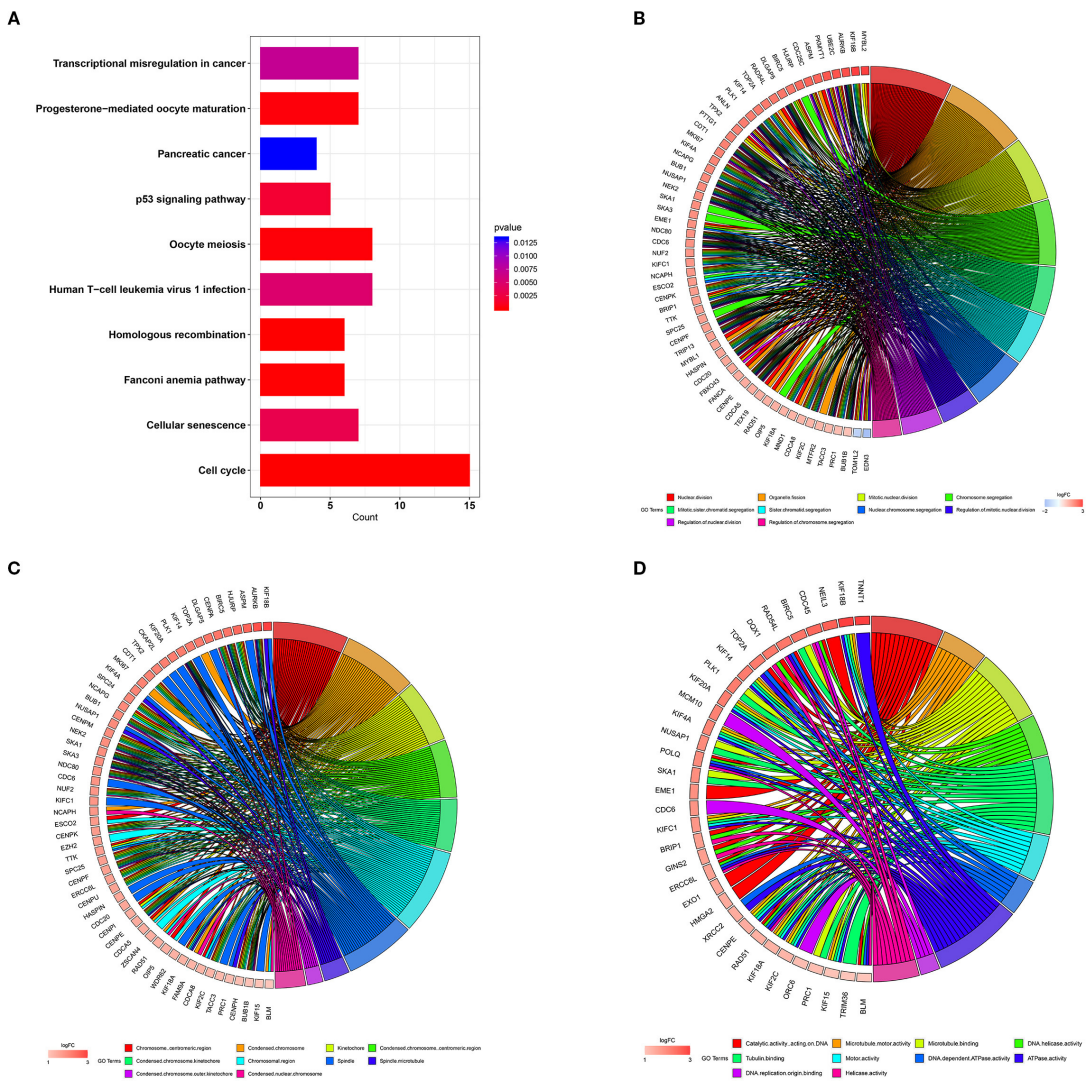
Functional annotation for pink module. **(A)** The bar plot of top 10 most significant signaling pathways identified by KEGG pathway enrichment analysis. The chord diagram of top 10 most significant GO terms and related genes in biological process **(B)** cell component **(C)** and molecular function **(D)**. KEGG, Kyoto Encyclopedia of Genes and Genomes; GO, gene ontology.

In order to study the function of the distant metastasis-related pink module, we performed a functional annotation analysis of all genes in the pink module. The top 10 enriched KEGG pathways were presented in Figure 4A, including Human T-cell leukemia virus 1 infection, cell cycle, p53 signaling pathway, transcriptional misregulation in cancer, and homologous recombination. The top 10 enriched GO terms were shown in Figures 4B–D, containing nuclear division, organelle fission, and chromosome segregation in the biological process category, spindle, chromosomal region, chromosome centromeric region in the cell component category, DNA replication origin binding, DNA-dependent ATPase activity, and tubulin binding in the molecular function category. The detailed information of functional annotation is listed in Supplementary Table 2, and the threshold is at *p* < 0.05.

### Identification and Verification of Hub mRNAs

**Figure 5:**
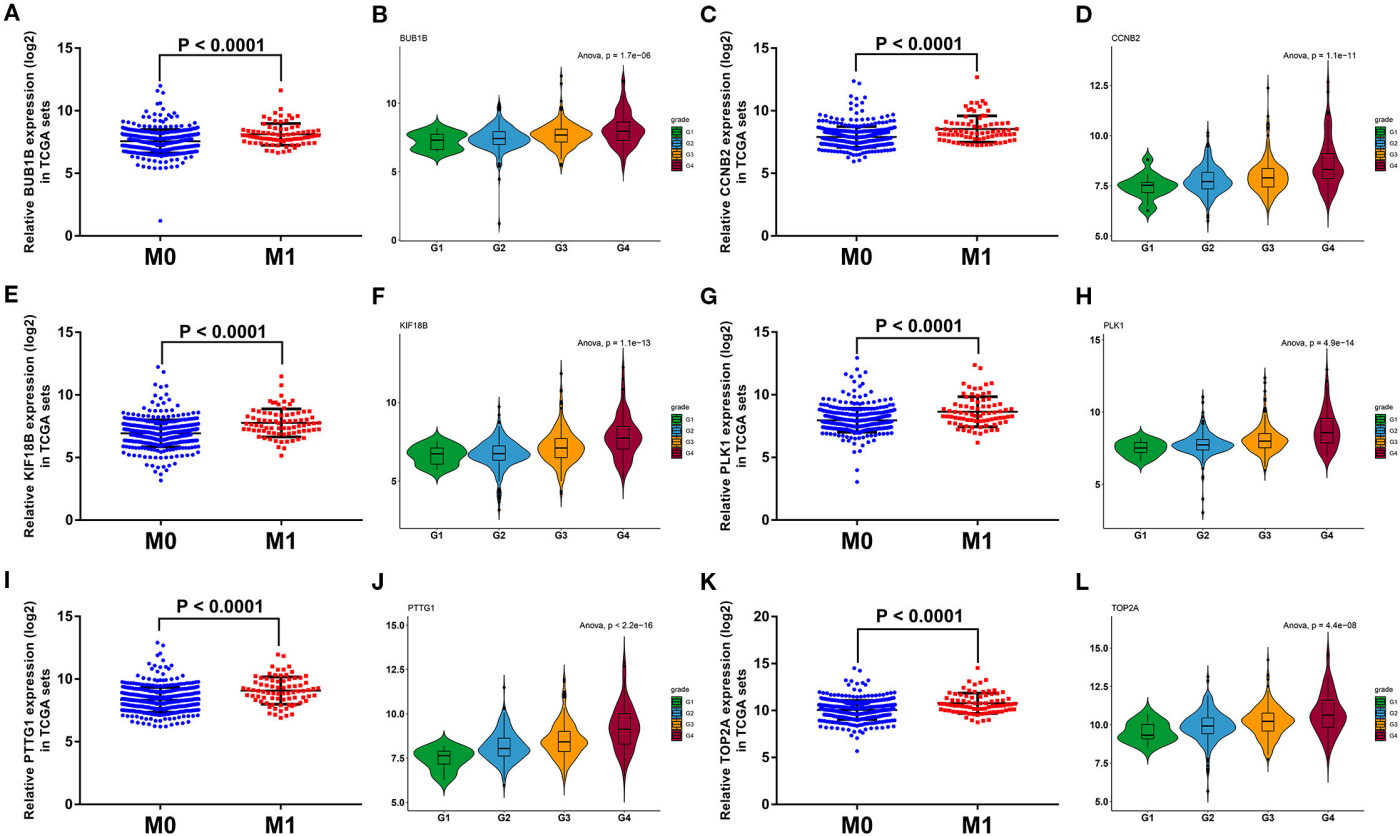
Grade and distant metastasis expression analyses of six hub mRNAs in the TCGA database. Expression analyses of BUB1B **(A)**, CCNB2 **(C)**, KIF18B **(E)**, PLK1 **(G)**, PTTG1 **(I)**, TOP2A **(K)** between distant metastasis samples (M1) and non-distant metastasis samples (M0). Expression patterns of BUB1B **(B)**, CCNB2 **(D)**, KIF18B **(F)**, PLK1 **(H)**, PTTG1 **(J)**, TOP2A **(L)** in different grades. The value of *p* < 0.05 iss considered statistically significant. TCGA, the cancer genome atlas; mRNAs, messenger RNAs.

To further screen the most significant hub genes, we conducted a series of analyses in the pink module. First, we visualized the WGCNA network of pink modules using Cytoscape 3.6.1, and 54 WGCNA hub genes with the node degree >200 were screened for next analyses (Figure 3F). Secondly, we uploaded these 54 genes to the STRING database for PPI network analysis. Under the threshold of a minimum interaction score >0.99, 33 PPI hub mRNAs were identified (Figure 3G, Supplementary Table 3). Thirdly, survival analysis was performed to these 33 PPI hub mRNAs in TCGA set and 30 mRNAs were significantly positively associated with poor prognosis in patients with ccRCC (Supplementary Figure 2). Fourthly, we conducted expression analysis to these 30 mRNAs separately in stage, grade, distant metastasis, ccRCC, and normal tissues and eventually obtained six hub mRNAs (BUB1B, CCNB2, KIF18B, PLK1, PTTG1, and TOP2A) that were not only upregulated in the samples of distant metastasis but were also positively significantly associated with higher grades in patients with ccRCC (Figure 5). Furthermore, the six hub mRNAs were also positively significantly associated with higher stages in patients with ccRCC (Supplementary Figures 3A–F) and highly expressed in tumor tissues (Supplementary Figures 3G–L). The detailed screening process of hub mRNAs is shown in Supplementary Table 4.

To verify the prognostic value of six hub mRNAs, we performed a survival analysis in the GEPIA database. The results showed that all six hub genes were significantly predictive of OS and DFS in patients with ccRCC (Supplementary Figures 3M–X). Meanwhile, the external validation data set, GSE53757 was used to confirm the expression patterns of six hub mRNAs. Consistent with our previous analyses in TCGA set, all six hub mRNAs were significantly positively associated with the pathological stage of patients with ccRCC (Supplementary Figures 4A–F) and were upregulated in ccRCC tissues (Supplementary Figures 4G–L).

### Screening of 3-Key miRNAs Linked to Distant Metastasis of ccRCC

**Figure 6:**
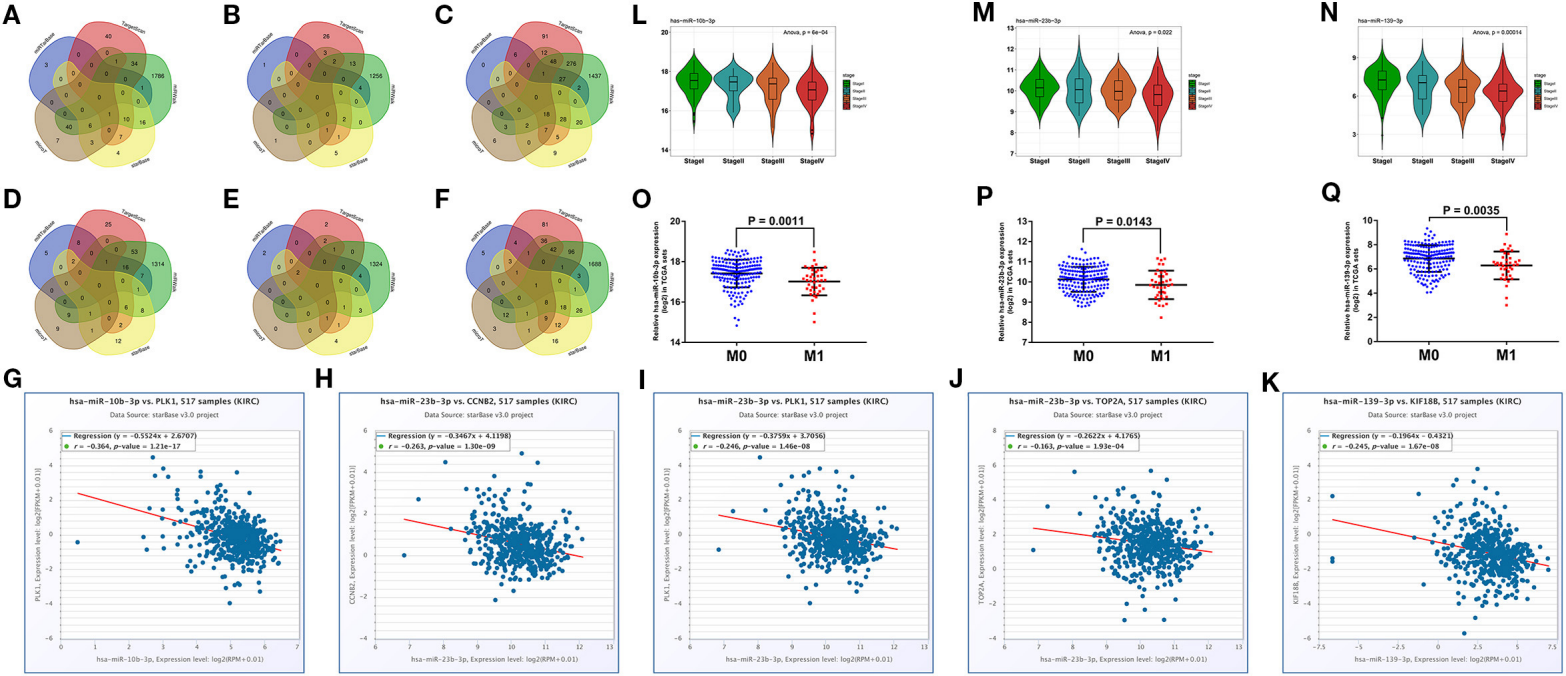
Prediction and screening of upstream potential miRNAs for six hub mRNAs. Venn diagram of target miRNAs for BUB1B **(A)**, CCNB2 **(B)**, KIF18B **(C)**, PLK1 **(D)**, PTTG1 **(E)**, and TOP2A **(F)**. **(G)** The expression correlation between hsa-miR-10b-3p and PLK1 in ccRCC. **(H)** The expression correlation between hsa-miR-23b-3p and CCNB2 in ccRCC. **(I)** The expression correlation between hsa-miR-23b-3p and PLK1 in ccRCC. **(J)** The expression correlation between hsa-miR-23b-3p and TOP2A in ccRCC. **(K)** The expression correlation between hsa-miR-139-3p and KIF18B in ccRCC. Expression analyses of hsa-miR-10b-3p **(L)**, hsa-miR-23b-3p **(M)**, hsa-miR-139-3p **(N)** among different stages. Expression pattern of hsa-miR-10b-3p **(O)**, hsa-miR-23b-3p **(P)**, hsa-miR-139-3p **(Q)** between distant metastasis samples (M1) and non-distant metastasis samples (M0). The value of *p* < 0.05 was considered statistically significant.

We predicted the upstream miRNAs of six hub mRNAs through five online databases as mentioned above and selected genes that co-occurred in two or more databases for further analysis (Figures 6A–F). In view of miRNA functional mechanism and oncogenic roles of six hub mRNAs, upstream miRNAs of the six hub mRNAs could be tumor suppressive genes. Therefore, all miRNAs–mRNAs pairs were selected for expression correlation analysis, and 45 miRNAs were significantly negatively correlated with targeting hub mRNAs. To evaluate the prognostic value of these miRNAs, we performed survival analysis for all of 45 miRNAs, and 18 miRNAs were significantly associated with good prognosis in patients with ccRCC (Supplementary Figure 5). Subsequently, we compared the expression level between ccRCC samples and normal samples for these 18 prognosis-related miRNAs, and 11 tumor suppressor miRNAs significantly downregulated in ccRCC tissues were identified (Supplementary Figure 6). Finally, we conducted expression analyses in stage and distant metastasis in these 11 miRNAs. The results indicated that the three key miRNAs (hsa-miR-10b-3p, hsa-miR-23b-3p, and hsa-miR-139-3p) were not only significantly negatively related to the expression of four targeting hub mRNAs (CCNB2, KIF18B, PLK1, and TOP2A, Figures 6G–K) and higher pathological stages in patients with ccRCC (Figures 6L–N) but also significantly low-expressed in distant metastasis tissues (Figures 6O–Q). The detailed screening process of key miRNAs is displayed in Supplementary Table 5.

### Upstream Distant Metastasis-Related LncRNAs of 3-Key miRNAs

**Figure 7:**
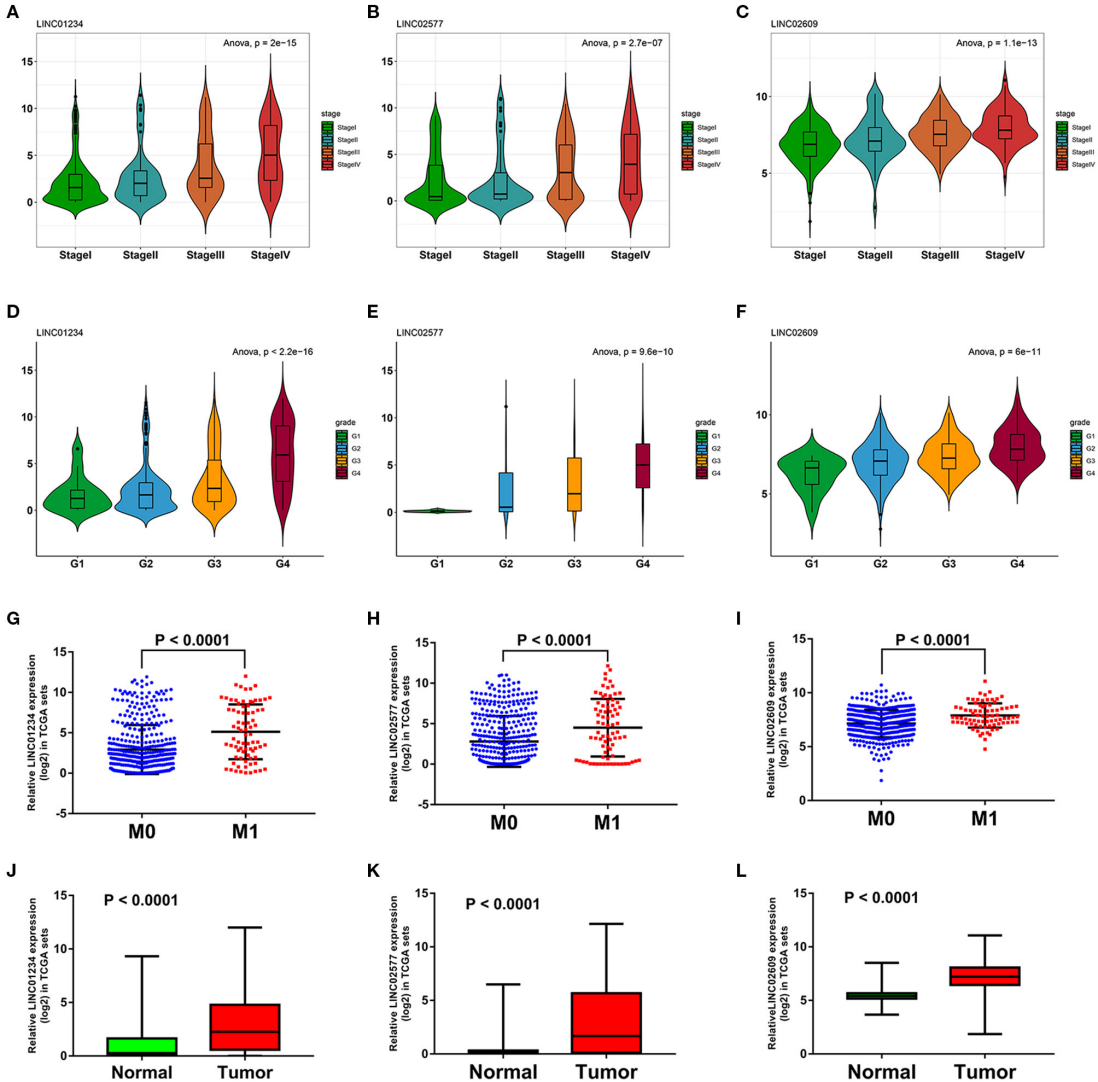
Expression analyses of three key lncRNAs in stage, grade, distant metastasis, normal, and tumor tissues. Expression analyses of LINC01234 **(A)**, LINC02577 **(B)**, LINC02609 **(C)** among different stages. Expression patterns of LINC01234 **(D)**, LINC02577 **(E)**, LINC02609 **(F)** in various grades. Expression analyses of LINC01234 **(G)**, LINC02577 **(H)**, LINC02609 **(I)** between distant metastasis samples (M1) and non-distant metastasis samples (M0). Expression analyses of LINC01234 **(J)**, LINC02577 **(K)**, LINC02609 **(L)** between normal tissues and tumor tissues. The value of *p* < 0.05 was considered statistically significant. lncRNAs, Long non-coding RNAs.

**Figure 8:**
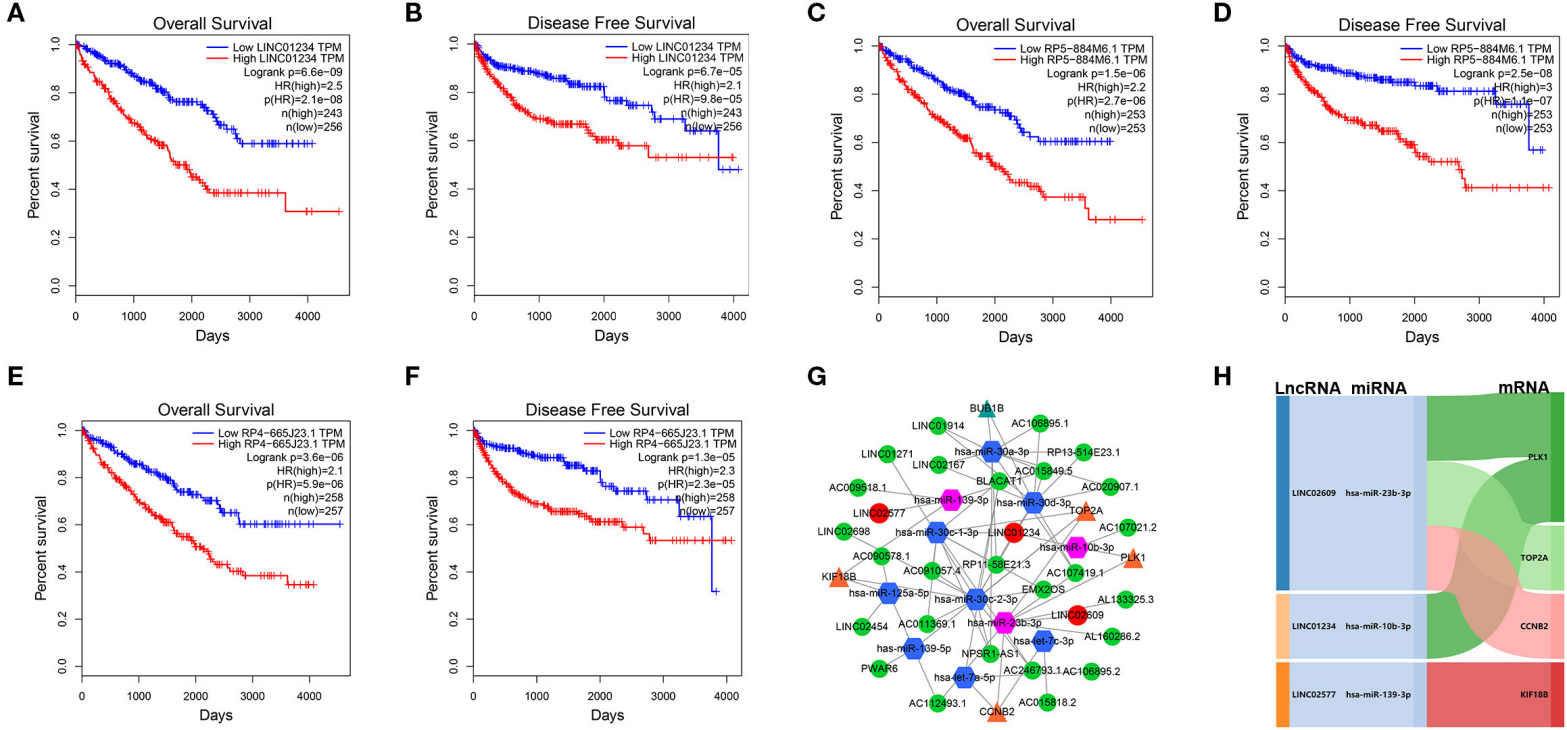
Survival analyses of key lncRNAs and ceRNA network construction. Survival analyses of LINC01234 **(A)** LINC02577/RP5-884M6.1 **(C)**, LINC02609/RP4-665J23.1 **(E)** in OS, and survival analyses of LINC01234 **(B)**, LINC02577/RP5-884M6.1 **(D)**, LINC02609/RP4-665J23.1 **(F)** in DFS. **(G)** ceRNA network of the 11 tumor suppressor miRNAs and their interactions in pink module. The green circle represents non-key lncRNA, the red circle represents key lncRNA, the blue hexagon stands for prognostic miRNA, the red hexagon stands for prognostic and distant metastatic miRNAs, the yellow triangle means hub mRNAs that are related to key miRNAs, and the green triangle means hub mRNAs that are only associated with prognostic miRNAs. **(H)** The core ceRNA network associated with distant metastasis and prognosis of patients with ccRCC was presented by Sankey diagram. The value of *p* < 0.05 was considered statistically significant. lncRNAs, Long non-coding RNAs; ceRNA, competitive endogenous RNA; OS, overall survival; DFS, disease free survival.

We utilized LncBase database to predict the upstream potential of lncRNAs that may be combined with the above 11 tumor suppressor miRNAs. To obtain distant metastasis-associated lncRNAs, an intersection was taken between the predicted lncRNAs and genes in the distant metastasis-associated pink module, and a total of 26 lncRNAs were finally obtained. To fully screen the distant metastasis-related lncRNAs, we chose the upstream 10 lncRNAs of three key miRNAs for subsequent analysis (Supplementary Figures 7A–C). Based on ceRNA mechanism, these lncRNAs may function as oncogenes in ccRCC and could negatively regulate downstream key miRNAs. Therefore, we conducted expression correlation analysis for these 10 lncRNAs, and found that nine lncRNAs were significantly negatively correlated with the downstream miRNAs (Supplementary Figures 7D–M). Subsequently, the expression differences of nine lncRNAs among different clinical traits including stage, grade, distant metastasis, and normal and tumor tissues were explored in the TCGA set. As a result, three key lncRNAs (LINC01234, LINC02577, and LINC02609) were identified, which not only significantly expressed higher in advanced stages and grades than in the early stages and grades (Figures 7A–F) but significantly upregulated in the tissues of tumor and distant metastasis than in the normal and non-distant metastasis control (Figures 7G–L). Finally, survival analysis indicated that the three lncRNAs were significantly associated with the poor prognosis of both OS and DFS in patients with ccRCC (Figures 8A–F). The detailed screening process of key lncRNAs is shown in Supplementary Table 6.

### Construction of ceRNA Regulatory Network in ccRCC

Eleven tumor suppressor miRNAs and their interactions in the pink module were used to construct the lncRNA–miRNA-mRNA network (Figure 8G). To further explore the potential mechanism of distant metastasis in ccRCC, all of the interactional key genes including the three key lncRNAs (LINC01234, LINC02577, and LINC02609), three key miRNAs (hsa-miR-10b-3p, hsa-miR-23b-3p, and hsa-miR-139-3p), and four hub mRNAs (CCNB2, KIF18B, PLK1, and TOP2A) in the network were extracted to form a core ceRNA network, and visualized by the Sankey diagram, in which all genes were significantly associated with distant metastasis and prognosis in patients with ccRCC (Figure 8H).

### The Distant Metastasis-Related LncRNA Signature as a Prognostic Indicator Independent of Other Clinical Characteristics

**Figure 9:**
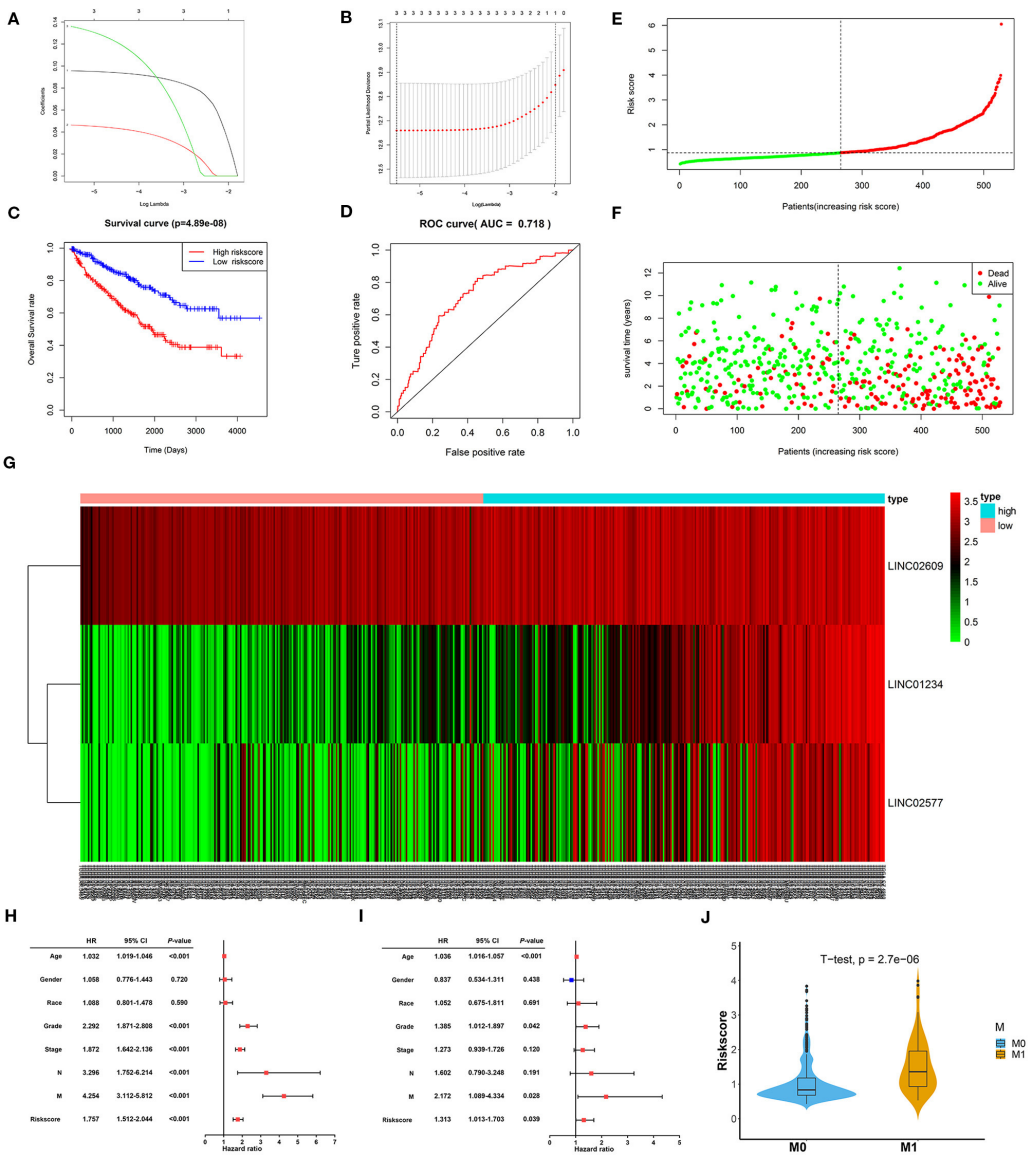
Constructionand assessment of the 3-lncRNA prognostic signature. **(A)** The coefficients in the LASSO regression for key lncRNAs. **(B)** Cross-validation for tuning parameter selection in the LASSO regression. **(C)** Kaplan–Meier curve to compare the OS between high- and low-risk score groups. **(D)** ROC curves showing predictive value of the 3-lncRNA prognostic signature. **(E)** Distribution of the risk scores based on 3-lncRNA prognostic signature in patients with ccRCC. **(F)** Survival status and time of patients with ccRCC and different risk scores in the TCGA set. The patients with ccRCC were separated into high- and low-risk groups according to the median of risk score. Red dots mean non-survivors, and green dots mean survivors. **(G)** Heatmap and hierarchical clustering of the three lncRNAs in the prognostic signature. The right longitudinal axis means the name of key lncRNAs, and the left longitudinal axis means the clustering situation of key lncRNAs. Red and green, respectively indicate upregulation and downregulation of lncRNAs. The upper horizontal axis denotes patients with ccRCC, while the blue means patients with high-risk ccRCC and the pink means patients with low-risk ccRCC. **(H)** Univariate Cox regression analysis of correlations between the risk score for OS and clinical phenotype. **(I)** Multivariate Cox regression analysis of correlations between the risk score for OS and clinical phenotype. **(J)** The distribution of risk score between distant metastasis patients (M1) and non-distant metastasis patients (M0). The value of *p* < 0.05 was considered statistically significant. LASSO, least absolute shrinkage and selection operator; ROC, receiver operating characteristic; OS, overall survival; ccRCC, clear cell renal cell carcinoma.

To further verify the prognostic potential of these three distant metastasis-associated lncRNAs, a univariate Cox proportional hazards regression analysis was performed. As a result, all three lncRNAs were significantly associated with poor prognosis in patients with ccRCC (*p* < 0.0001, Table 3). Then, the key lncRNAs were further evaluated by LASSO regression analysis, and we repeated the process 1,000 times to avoid over-fitting in the prognostic signature. The result displayed that all of the three key lncRNAs could be included for further analysis (Figures 9A,B). Subsequently, through multivariate Cox regression analysis (Table 3), we built the three lncRNA prognostic signature to predict OS as follows:

**Table 3:** Univariate and multivariate cox regression of key lncRNAs.

**Univariate cox regression**				**Multivariate cox regression**			
**Gene name**	**HR**	**95% CI**	***P-*value**	**Coef**	**HR**	**95% CI**	***P*-value**
LINC01234	1.151	1.11–1.20	<0.0001	0.0977	1.103	1.05–1.16	0.0002
LINC02577	1.122	1.08–1.17	<0.0001	0.0464	1.048	1.00–1.10	0.0759
LINC02609	1.304	1.15–1.48	<0.0001	0.1350	1.145	1.00–1.31	0.0458

Risk score = (0.0977 × expression level of LINC01234) + (0.1350 × expression level of LINC02609) + (0.0464 × expression level of LINC02577).

Of note, LINC01234 is all the most closely associated with prognostic signature (*p* = 0.00022). The OS rate of high-risk patients was significantly lower than that of low-risk patients (Figure 9C). Furthermore, the area under ROC curve was 0.718 (Figure 9D), showing a high predictive ability of the prognostic signature. Finally, all 529 patients in the TCGA set were divided into high-risk (*n* = 264) and low-risk groups (*n* = 265) according to the median value of the risk score. Risk curve and scatterplot separately displayed the risk score and survival status of each patient with ccRCC (Figures 9E,F). Patients in the low-risk group had lower mortality than patients in the high-risk group. The heatmap showed the expression level of the prognostic signature in patients with ccRCC, indicating that the three key lncRNAs were obviously highly expressed in high-risk patients with ccRCC (Figure 9G). Additionally, the univariate and multivariate Cox regression analyses of clinical characteristics displayed that age, grade, M stage, and the risk score were the independent prognostic factors (Figures 9H,I). The risk score of patients with distant metastasis was significantly higher than that of patients with non-distant metastasis, which indicated that the patients with higher risk score were more likely to develop distant metastasis compared to the patients with lower risk score (Figure 9J).

### GSEA of the Distant Metastasis-Related LncRNA Signature

**Figure 10:**
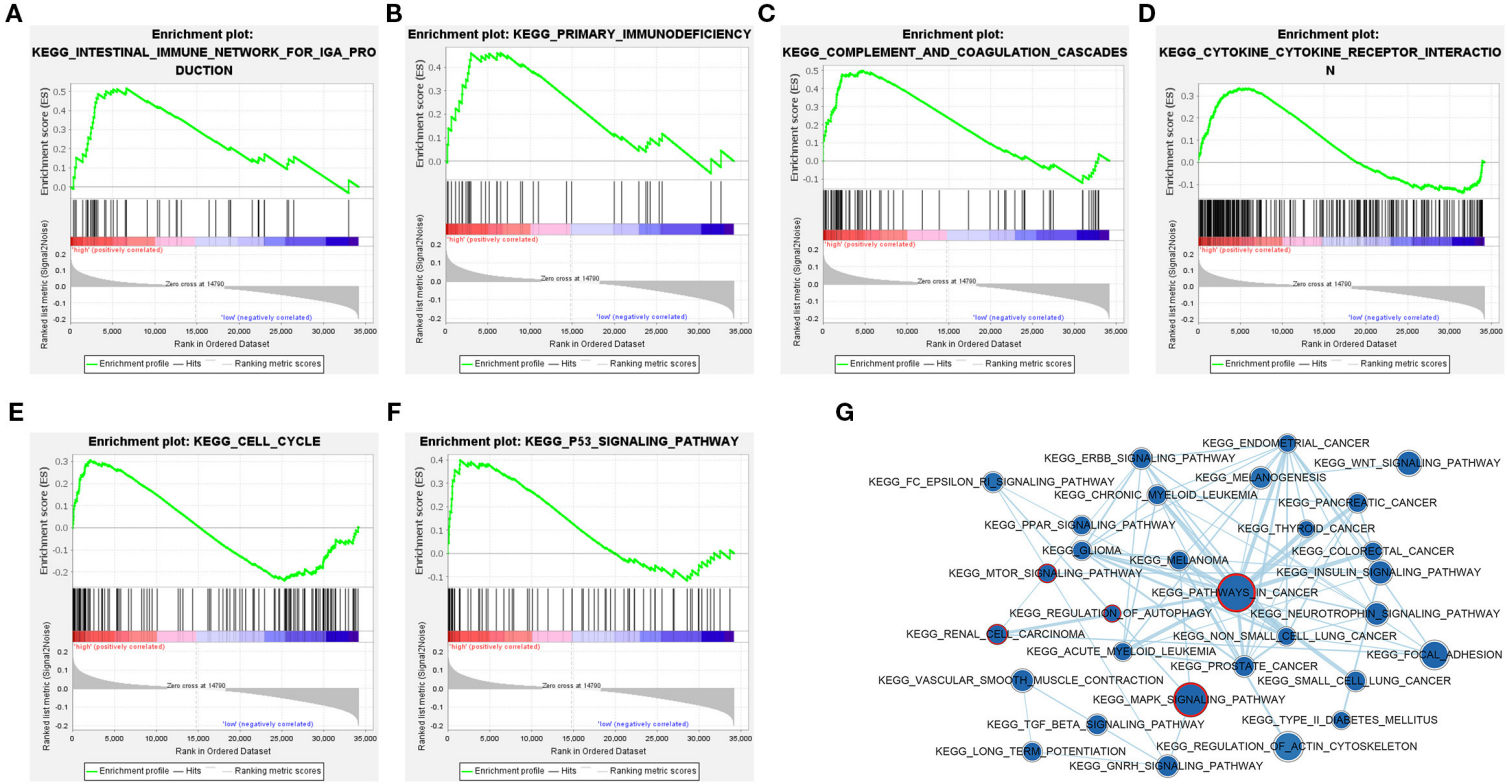
Gene set enrichment analysis (GSEA) identified gene sets significantly enriched in the phenotype of high- or low-risk patients based on the distant metastasis-related lncRNA prognosis signature. **(A–F)** GSEA results displayed significant enrichment of immune-related signaling pathways in patients with high-risk ccRCC. **(G)** The GSEA network indicated that cancer-related and autophagy-related signaling pathways are significantly enriched in patients with low-risk ccRCC. The nodes represent signaling pathways, and the larger the nodes, the more genes are enriched in the signaling pathway. Edges stand for mutual overlap, and the wider the edges, more genes overlap among the gene sets. The representative cancer- and autophagy-related signaling pathways are marked with red outline. The value of *p* < 0.05 is considered statistically significant. GSEA, gene set enrichment analysis; ccRCC, clear cell renal cell carcinoma.

In order to explore the potential pathways of the 3-lncRNA prognostic signature, we performed GSEA to the signature in the TCGA set. The result indicated that the patients with high-risk score were mainly associated with immunoregulatory pathways, such as primary immunodeficiency, intestinal immune network for lgA production, complement and coagulation cascades, cytokine–cytokine receptor interaction, cell cycle, and p53 signaling pathway (Figures 10A–F). Autophagy-related and cancer-related pathways were mainly enriched in low-risk patients, including regulation of autophagy, mTOR signaling pathway, renal cell carcinoma, pathways in cancer, and MAPK signaling pathway (Figure 10G), indicating that the autophagy was mainly involved in patients with low-risk ccRCC. The top eight signaling pathways in high- and low-risk groups based on GSEA are shown in Table 4. These results suggested that a high-risk score in patients with ccRCC may be linked to impaired immune function, while a low risk score in patients with ccRCC might be correlated with enhanced autophagy.

**Table 4:** GSEA based on the 3-lncRNA prognostic signature.

**Group**	**Pathway**	**Enrichment score**	**NOM *p*-value**	**FDR *q*-value**
High risk	KEGG_COMPLEMENT_AND_COAGULATION_CASCADES	0.500	0.000	0.004
	KEGG_INTESTINAL_IMMUNE_NETWORK_FOR_IGA_PRODUCTION	0.515	0.000	0.005
	KEGG_P53_SIGNALING_PATHWAY	0.399	0.000	0.034
	KEGG_CYTOKINE_CYTOKINE_RECEPTOR_INTERACTION	0.333	0.000	0.073
	KEGG_CELL_CYCLE	0.305	0.000	0.130
	KEGG_PRIMARY_IMMUNODEFICIENCY	0.461	0.007	0.035
	KEGG_PROTEASOME	0.459	0.010	0.028
	KEGG_DNA_REPLICATION	0.400	0.024	0.122
Low risk	KEGG_MAPK_SIGNALING_PATHWAY	−0.426	0.000	0.083
	KEGG_PATHWAYS_IN_CANCER	−0.408	0.001	0.102
	KEGG_RENAL_CELL_CARCINOMA	−0.501	0.002	0.027
	KEGG_MTOR_SIGNALING_PATHWAY	−0.526	0.002	0.026
	KEGG_ERBB_SIGNALING_PATHWAY	−0.495	0.006	0.028
	KEGG_SMALL_CELL_LUNG_CANCER	−0.476	0.007	0.050
	KEGG_REGULATION_OF_AUTOPHAGY	−0.524	0.040	0.075
	KEGG_FC_EPSILON_RI_SIGNALING_PATHWAY	−0.433	0.040	0.126

### Validation of Distant Metastasis-Related LncRNA Signature in Tissues and Cell Lines

**Figure 11:**
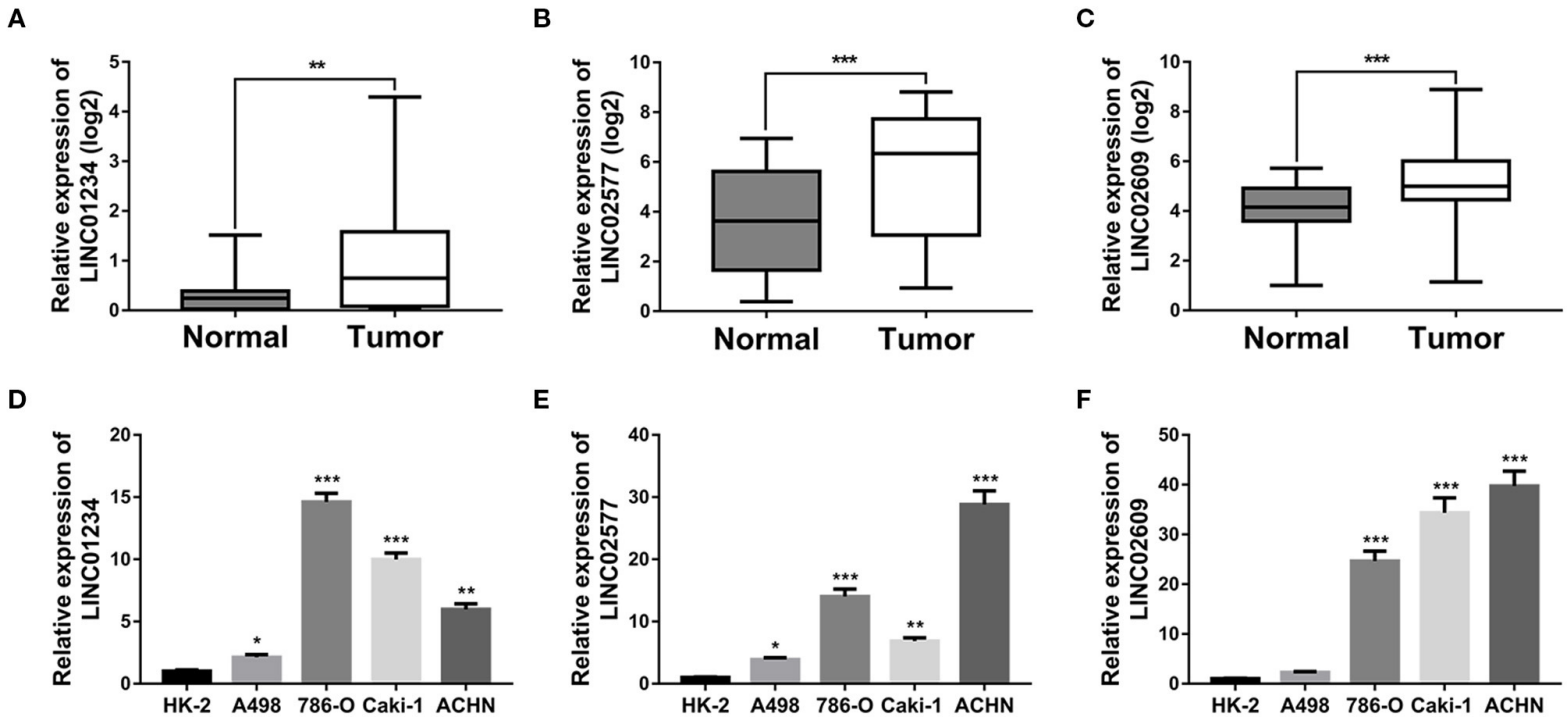
Quantitative real-time (qRT)-PCR validation of three key lncRNAs. Relative expression levels of LINC01234 **(A)**, LINC02577 **(B)** and LINC02609 **(C)** in 30 matched normal and ccRCC tissues harvested at The First Affiliated Hospital of Anhui Medical University. Relative expression levels of LINC01234 **(D)**, LINC02577 **(E)**, and LINC02609 **(F)** between normal renal tubular epithelial cell line and ccRCC cell lines was determined by qRT-PCR, where *p < 0.05, **p < 0.01, and ***p < 0.001. lncRNAs, long non-coding RNAs; qRT-PCR, quantitative real-time-PCR.

To verify the expression patterns of the 3-lncRNA prognostic signature, the expression level of the three lncRNAs was further verified by qRT-PCR, not only in 30 paired ccRCC/normal tissue specimens that were collected at our institution but in one normal renal tubular epithelial cell line (HK-2) and 4 ccRCC cell lines (A498, 786-O, Caki-1, and ACHN). The results indicated that the three key lncRNAs in the prognostic signature were not only significantly expressed high in tumor tissues than in normal tissues (Figures 11A–C) but significantly upregulated in the cell lines of ccRCC compared to the cell line of normal renal tubular epithelial cells (Figures 11D–F), which is in agreement with the result of the database analysis from our study.

## Discussion

Clear cell renal cell carcinoma, one of the most common malignancies of the urinary system, is prone to recur and result in metastasis. Approximately 25–30% of patients with ccRCC already exhibited distant metastasis at the initial diagnosis (24). Although great progress has been made in the diagnosis and treatment of ccRCC, the 5-year survival rate of patients with ccRCC and distant metastasis remain very low (5). Therefore, the exploration of prognostic biomarkers with high sensitivity and specificity for distant metastasis in patients with ccRCC has become increasingly urgent. The dysregulation and mutation of lncRNAs could promote or inhibit the occurrence and metastasis of tumors, which makes them to be the novel prognostic biomarkers and promising therapeutic targets in cancers (7). At present, some lncRNAs have been found to play an important role in the prognosis of cancers, including CYTOR (25), CASC19 (26), IGFBP4-1 (27), and CDC6 (28). However, numerous studies indicated that the combined prognostic model of lncRNAs has a prominent advantage over the single lncRNA prognostic biomarker in terms of statistical stability. Although some combined prognostic models of lncRNAs have been identified in multiple types of cancers (29, 30), studies on combined prognostic signatures of lncRNAs in ccRCC are still very limited. Additionally, different from the other lncRNA signatures that were only associated with ccRCC prognosis (31, 32), the lncRNA signature we constructed was closely associated with distant metastasis in ccRCC and prognosis simultaneously. More importantly, a potential ceRNA regulatory network of the lncRNA signature was constructed, which all genes in the network could be regarded as the promising biomarkers that accurately predicted distant metastasis and prognosis of patients with ccRCC, achieving the precise identification of distant metastasis-associated ceRNA regulatory network in ccRCC for the first time.

In the present study, we first downloaded the transcriptome sequencing data of ccRCC and the corresponding clinical information from patients with ccRCC obtained from the database of TCGA. Unlike other similar studies that only used one package to identify DEGs (33, 34), we simultaneously utilized the R packages, such as “DESeq2” and “edgeR,” and finally obtained 11,034 DEGs after intersecting the results of the analysis. Subsequently, 23 gene co-expression modules were constructed using WGCNA. Among them, we identified the key pink module that was all the most closely associated with distant metastasis of ccRCC using the Pearson's correlation analysis. Interestingly, it was found that the pink module was also all the most closely associated with the pathological stage and histological grade of ccRCC, indicating that the pink module has an important clinical significance. After conducting a series of rigorous analyses, such as PPI analysis, survival analysis, expression analyses in stage, grade, and distant metastasis to genes in the pink module, a total of six hub mRNAs (BUB1B, CCNB2, KIF18B, PLK1, PTTG1, and TOP2A) that could predict distant metastasis and poor prognosis in patients with ccRCC were successfully identified. Subsequently, the GEO data set and GEPIA database were utilized for verification, which further confirmed that the six hub mRNAs were upregulated in the tissues of ccRCC, and were highly correlated with the poor prognosis and the pathological stages of patients with ccRCC. Moreover, some studies have also indicated that the six hub mRNAs could be used as promising biomarkers related to the metastasis or prognosis of cancer. For example, over-expression of BUB1B is associated with poor prognosis in patients with prostate cancer, and could promote proliferation and migration of prostate cancer cells (35); Wang et al. (36) proposed that miR-335-5p could regulate metastasis of lung adenocarcinoma by targeting CCNB2. Yan et al. (37) showed that KIF18B could act as a promoting factor for proliferation and metastasis of cutaneous melanoma. Fu et al. (38) found that the over-expression of PLK1 could induce oncogenic transformation of prostate epithelial cells, thereby driving the metastasis of prostate cancer. Song et al. (39) suggested that miR-144-3p could inhibit proliferation and metastasis of glioma cells by targeting TOP2A. Huang et al. (40) found that upregulation of PTTG1 was able to promote the growth and metastasis of hepatocellular carcinoma cells. These reports together with the results of our analyses further confirmed that the six hub mRNAs may function as oncogenes to play a crucial role in distant metastasis and poor prognosis in patients with ccRCC. MiRNAs can negatively regulate the expression of genes at the post-transcriptional level by interacting with MREs, thereby promoting or inhibiting the progression of cancer (41). Therefore, it is of great significance to explore the miRNAs targeting the above six hub mRNAs. First, five online databases were utilized to predict the potential miRNAs. According to the action mechanism of miRNAs in the ceRNA hypothesis, we speculated that the above miRNAs may function as tumor suppressor genes in ccRCC. Based on this inference, we screened three key miRNAs (hsa-miR-10b-3p, hsa-miR-23b-3p, and hsa-miR-139-3p) targeting four hub mRNAs (CCNB2, KIF18B, PLK1, and TOP2A) using the correlation analysis, survival analysis, expression analyses in stage, distant metastasis, tumor, and normal tissues. It was reported that the three key miRNAs played a key regulatory role in the oncogenesis and development of human cancers. For instance, Yang et al. (42) constructed a prediction model of five-miRNAs, including hsa-miR-10b-3p that functions as the independent prognostic factor in colorectal cancer; Zhou et al. (43) found that patients with higher hsa-miR-23b-3p expression had better outcomes than those with lower expression of hsa-miR-23b-3p in colorectal cancer. Xu et al. (44) demonstrated that over-expression of hsa-miR-139-3p could significantly inhibit the proliferation, migration, and invasion of HeLa cells in cervical cancer, *in vitro* and could significantly suppress the growth of cervical tumor *in vivo*. All of these reports further supported the accuracy of the results of the current study.

In addition to miRNAs, lncRNAs can function as ceRNAs to interact with mRNAs by competitively binding to specific miRNAs and thus regulate various human diseases including cancer (45). Therefore, we utilized LncBase database to predict lncRNAs that potentially bind to the 3-key miRNAs. To explore the potential molecular mechanism of distant metastasis in ccRCC, we took the intersection of the predicted lncRNAs with genes in the distant metastasis-associated pink module and obtained 10 lncRNAs. According to the ceRNA hypothesis (9), lncRNAs could function as ceRNAs to negatively regulate miRNAs, which means that the expression trend of lncRNAs may be opposite to that of miRNAs. Therefore, we performed a correlation analysis to the resulting 10 lncRNAs based on the above relationship. Subsequently, we conducted a series of strict analyses, such as survival analysis, expression analyses in stage, grade, distant metastasis, normal, and tumor tissues to the screened lncRNAs, and finally obtained three key lncRNAs with significant distant metastasis and prognosis potential (LINC01234, LINC02577, and LINC02609). Among them, LINC01234 has been reported to be involved in the biological processes of some cancers. For instance, Chen et al. (46) identified that LINC01234 was an oncogenic lncRNA that could promote proliferation and inhibit apoptosis of gastric cancer cells through miR-204-5p-CBFB axis. Lin et al. (47) found that LINC01234 was significantly upregulated in colon cancer, and promoted the proliferation of colon cancer cells by competitively combining miR-642a-5p. Chen et al. (48) demonstrated that LINC01234 was upregulated in human non-small cell lung cancer (NSCLC) and was closely associated with the metastasis and poor prognosis of NSCLC. However, there are very few reports on LINC01234 in ccRCC. Only Zhang et al. (49) constructed a risk score model of five lncRNAs including LINC01234 in ccRCC. More importantly, there is no relevant report on LINC01234 in the distant metastasis of ccRCC. Of note, LINC02577 and LINC02609 have not yet been reported in human cancers. Therefore, it is needed to conduct more studies on the three lncRNAs to explore their potential action mechanism in the development of tumors (especially ccRCC). Subsequently, we performed LASSO and Cox regression analyses to the above three lncRNAs, and the results further displayed that they were closely associated with poor prognosis in patients with ccRCC. Therefore, we constructed a risk score model in ccRCC using the above three lncRNAs to assess the prognosis of each patient. According to the median value of the risk score, we divided these patients into high- and low-risk groups. The results showed that the patients in the high-risk group have higher mortality and worse prognosis compared to the patients in the low-risk group. Finally, we evaluated the risk score model of lncRNAs using ROC curve in the entire TCGA data set, and the results showed that this model owned good predictive value. Furthermore, compared to the other clinical traits (age, gender, race, grade, stage, N stage, and M stage) of patients with ccRCC, the 3-lncRNA signature was an independent factor predicting the overall survival of patients with ccRCC. Additionally, GSEA results showed that the immune-related pathways were mainly enriched in the high-risk group, whereas the autophagy- and cancer-related pathways were mainly enriched in the low-risk group. These results revealed that the disorder of immune may be associated with the poor prognosis of patients with ccRCC. More importantly, the results also suggested that the three lncRNAs could promote distant metastasis of ccRCC by regulating autophagy, which is consistent with our previous findings (50). Finally, we took the four hub mRNAs (CCNB2, KIF18B, PLK1, and TOP2A), three key miRNAs (hsa-miR-10b-3p, hsa-miR-23b-3p, and hsa-miR-139-3p) and three key lncRNAs (LINC01234, LINC02577, and LINC02609) screened above together to construct a ceRNA network that could regulate the distant metastasis and prognosis in ccRCC. Compared to other similar reports in which only some genes play crucial molecular functions (51, 52), all genes in the ceRNA network are closely related to the distant metastasis and prognosis of patients with ccRCC. Moreover, the results of qRT-PCR at the histological and cellular levels showed that three key lncRNAs in the ceRNA network were significantly upregulated in the tissues and cell lines of ccRCC compared to that in the normal tissues and renal tubular epithelial cell, which further confirmed the accuracy of our previous analysis on bioinformatics on the clinical and experimental levels.

In conclusion, based on the three upregulated lncRNAs in ccRCC, we identified a distant metastasis-related signature that could independently predict the overall survival of patients with ccRCC. Furthermore, the potential ceRNA regulatory network of the three key lncRNAs was accurately constructed, and all genes in the network could be the predicted factors for the distant metastasis and prognosis in ccRCC, providing a new sight on the mechanism underlying the distant metastasis of ccRCC. The distant metastasis-related lncRNAs in the network could be used as reliable prognosis biomarkers and promising therapeutic targets for ccRCC, but in the future, further studies are still needed to verify the stability of this prognostic signature.

## Data Availability Statement

The original contributions presented in the study are included in the article/Supplementary Material, further inquiries can be directed to the corresponding author/s.

## Ethics Statement

The studies involving human participants were reviewed and approved by Ethics Committee of the First Affiliated Hospital of Anhui Medical University. The patients/participants provided their written informed consent to participate in this study.

## Author Contributions

YS and TZ conceived, designed the study, and writing of the manuscript. TZ acquired and analyzed the data. CL supervised and analyzed the results. JT and SF collected the tissue samples and cultured cells. YS performed experiments. LZ and JZ contributed analysis tools. All authors reviewed and approved the submitted manuscript.

## Conflict of Interest

The authors declare that the research was conducted in the absence of any commercial or financial relationships that could be construed as a potential conflict of interest.

## Supplementary Material

Supplementary Figure 1 Construction of co-expression modules and validation of pink module by WGCNA. **(A)** The cluster dendrogram of ccRCC samples. Analysis of network topology for different soft-thresholding powers in scale independence **(B)** and mean connectivity **(C)**. **(D)** Cluster dendrogram of all DEGs in the TCGA set. Each branch in the dendrogram represents a single gene, and every color below indicates a co-expression gene module. **(E)** The heatmap describes the TOM among genes based on divided co-expression modules. About 5,000 randomly selected genes were grouped into different modules, which were shown in different color codes below the cluster dendrogram. Light yellow displays a high degree of topological overlap, while dark red indicates a low degree of topological overlap. Scatter plots of GS for age **(F)**, gender **(G)**, and race **(H)** vs. MM in pink module. The value of *p* < 0.05 was considered statistically significant. WGCNA, weighted gene co-expression network analysis; ccRCC, Clear cell renal cell carcinoma; TOM, topological overlap matrix; DEGs, differentially expressed genes; GS, gene significance; MM, module membership.

Supplementary Figure 2 Survival analyses of 33 PPI hub mRNAs in the TCGA set. The value of *p* < 0.05 was considered statistically significant. PPI, protein–protein interaction; TCGA, the cancer genome atlas.

Supplementary Figure 3 Expression analyses and survival validation of six hub mRNAs. Expression analyses of BUB1B **(A)**, CCNB2 **(B)**, KIF18B **(C)**, PLK1 **(D)**, PTTG1 **(E)**, and TOP2A **(F)** in different stages. Expression analyses of BUB1B **(G)**, CCNB2 **(H)**, KIF18B **(I)**, PLK1 **(J)**, PTTG1 **(K)**, and TOP2A **(L)** between normal tissues and tumor tissues. OS validation of TOP2A **(M)**, BUB1B **(N)**, CCNB2 **(O)**, PTTG1 **(P)**, PLK1 **(Q)**, and KIF18B **(R)** in GEPIA database. DFS validation of TOP2A **(S)**, BUB1B **(T)**, CCNB2 **(U)**, PTTG1 **(V)**, PLK1 **(W)**, and KIF18B **(X)** in GEPIA database. The value of *p* < 0.05 was considered statistically significant. OS, overall survival; DFS, disease free survival; GEPIA, Gene Expression Profiling Interactive Analysis.

Supplementary Figure 4 Expression pattern validation in GEO dataset. Expression analyses of BUB1B **(A)**, CCNB2 **(B)**, KIF18B **(C)**, PLK1 **(D)**, PTTG1 **(E)**, TOP2A **(F)** among different stages. Expression levels of BUB1B **(G)**, CCNB2 **(H)**, KIF18B **(I)**, PLK1 **(J)**, PTTG1 **(K)**, TOP2A **(L)** between tumor tissues and normal tissues. The value of *p* < 0.05 was considered statistically significant. GEO, Gene expression omnibus.

Supplementary Figure 5 Target miRNAs associated with overall survival. The value of *p* < 0.05 was considered statistically significant. miRNAs, micro RNAs.

Supplementary Figure 6 Expression levels of 11 tumor suppressor miRNAs between ccRCC/KIRC tissues and normal tissues. The value of *p* < 0.05 was considered statistically significant. miRNAs, micro RNAs, ccRCC, Clear cell renal cell carcinoma.

Supplementary Figure 7 Screening of distant metastasis-related lncRNAs by intersection with pink module and expression correlation analyses. Venn diagram of target lncRNAs for hsa-miR-10b-3p **(A)**, hsa-miR-23b-3p **(B)**, and hsa-miR-139-3p **(C)**. **(D)** The expression correlation between hsa-miR-10b-3p and AC107021.2. **(E)** The expression correlation between hsa-miR-10b-3p and LINC01234. **(F)** The expression correlation between hsa-miR-23b-3p and AC107419.1. **(G)** The expression correlation between hsa-miR-23b-3p and AC112493.1. **(H)** The expression correlation between hsa-miR-23b-3p and AL133325.3. **(I)** The expression correlation between hsa-miR-23b-3p and AL160286.2. **(J)** The expression correlation between hsa-miR-23b-3p and LINC02609/AL590094.1. **(K)** The expression correlation between hsa-miR-139-3p and AC015849.5. **(L)** The expression correlation between hsa-miR-139-3p and AC009518.1. **(M)** The expression correlation between hsa-miR-139-3p and LINC02577. The value of *p* < 0.05 was considered statistically significant. lncRNAs, Long non-coding RNAs.

Supplementary Table 1 Number of genes in 23 co-expression modules.

Supplementary Table 2 Functional enrichment analyses of the genes in the distant metastasis-related pink module.

Supplementary Table 3 The combined score among nodes of PPI network in the co-expression pink module.

Supplementary Table 4 Hub mRNAs were screened by WGCNA, PPI, TCGA internal test, GEPIA external validation, and GEO external validation.

Supplementary Table 5 Screening for key miRNAs using correlation analysis, survival analysis, and expression analyses.

Supplementary Table 6 Screening for key lncRNAs using correlation analysis, expression analyses, and survival analysis.

## References

[1] Pichler R. Immune checkpoint inhibitors in uro-oncology: urgent call for biomarkers. Cancers. (2020) 12:2768. 10.3390/cancers12102768 32992444 PMC7601394

[2] Siegel RL Miller KD Jemal A. Cancer statistics, 2020. CA Cancer J Clin. (2020) 70:7–30. 10.3322/caac.21590 31912902

[3] Hsieh JJ Purdue MP Signoretti S Swanton C Albiges L Schmidinger M. Renal cell carcinoma. Nat Rev Dis Primers. (2017) 3:17009. 10.1038/nrdp.2017.9 28276433 PMC5936048

[4] Filippiadis D Mauri G Marra P Charalampopoulos G Gennaro N De Cobelli F. Percutaneous ablation techniques for renal cell carcinoma: current status and future trends. Int J Hyperthermia. (2019) 36:21–30. 10.1080/02656736.2019.1647352 31537160

[5] Li JK Chen C Liu JY Shi JZ Liu SP Liu B. Long noncoding RNA MRCCAT1 promotes metastasis of clear cell renal cell carcinoma via inhibiting NPR3 and activating p38-MAPK signaling. Mol Cancer. (2017) 16:111. 10.1186/s12943-017-0681-0 28659173 PMC5490088

[6] Ponting CP Oliver PL Reik W. Evolution and functions of long noncoding RNAs. Cell. (2009) 136:629–41. 10.1016/j.cell.2009.02.006 19239885

[7] Bhan A Soleimani M Mandal SS. Long noncoding RNA and cancer: a new paradigm. Cancer Res. (2017) 77:3965–81. 10.1158/0008-5472.CAN-16-2634 28701486 PMC8330958

[8] Tang S Li S Liu T He Y Hu H Zhu Y. MicroRNAs: emerging oncogenic and tumor-suppressive regulators, biomarkers and therapeutic targets in lung cancer. Cancer Lett. (2021) 502:71–83. 10.1016/j.canlet.2020.12.040 33453304

[9] Salmena L Poliseno L Tay Y Kats L Pandolfi PP. A ceRNA hypothesis: the rosetta stone of a hidden RNA language? Cell. (2011) 146:353–8. 10.1016/j.cell.2011.07.014 21802130 PMC3235919

[10] Yu D Ruan X Huang J Hu W Chen C Xu Y. Comprehensive analysis of competitive endogenous RNAs network, being associated with esophageal squamous cell carcinoma and its emerging role in head and neck squamous cell carcinoma. Front Oncol. (2019) 9:1474. 10.3389/fonc.2019.01474 32038997 PMC6985543

[11] Zhou J Guo H Liu L Hao S Guo Z Zhang F. Construction of co-expression modules related to survival by WGCNA and identification of potential prognostic biomarkers in glioblastoma. J Cell Mol Med. (2021) 25:1633–44. 10.21203/rs.3.rs-27332/v1 33449451 PMC7875936

[12] Ding C Han F Xiang H Xia X Wang Y Dou M. LncRNA CRNDE is a biomarker for clinical progression and poor prognosis in clear cell renal cell carcinoma. J Cell Biochem. (2018) 119:10406–14. 10.1002/jcb.27389 30129055

[13] Qu L Wang ZL Chen Q Li YM He HW Hsieh JJ. prognostic value of a long non-coding RNA signature in localized clear cell renal cell carcinoma. EurUrol. (2018) 74:756–63. 10.1016/j.eururo.2018.07.032 30143382

[14] Ritchie ME Phipson B Wu D Hu Y Law CW Shi W. limma powers differential expression analyses for RNA-sequencing and microarray studies. Nucleic Acids Res. (2015) 43:e47. 10.1093/nar/gkv007 25605792 PMC4402510

[15] Lun AT Chen Y Smyth GK. It's DE-licious: a recipe for differential expression analyses of RNA-seq experiments using quasi-likelihood methods in edgeR. Methods MolBiol. (2016) 1418:391–416. 10.1007/978-1-4939-3578-9_19 27008025

[16] Love MI Huber W Anders S. Moderated estimation of fold change and dispersion for RNA-seq data with DESeq2. Genome Biol. (2014) 15:550. 10.1186/s13059-014-0550-8 25516281 PMC4302049

[17] Langfelder P Horvath S. WGCNA: an R package for weighted correlation network analysis. BMC Bioinformatics. (2008) 9:559. 10.1186/1471-2105-9-559 19114008 PMC2631488

[18] Yu G Wang LG Han Y He QY. clusterProfiler: an R package for comparing biological themes among gene clusters. Omics. (2012) 16:284–7. 10.1089/omi.2011.0118 22455463 PMC3339379

[19] Shannon P Markiel A Ozier O Baliga NS Wang JT Ramage D. Cytoscape: a software environment for integrated models of biomolecular interaction networks. Genome Res. (2003) 13:2498–504. 10.1101/gr.1239303 14597658 PMC403769

[20] Szklarczyk D Gable AL Lyon D Junge A Wyder S Huerta-Cepas J. STRING v11: protein-protein association networks with increased coverage, supporting functional discovery in genome-wide experimental datasets. Nucleic Acids Res. (2019) 47:D607–13. 10.1093/nar/gky1131 30476243 PMC6323986

[21] Li JH Liu S Zhou H Qu LH Yang JH. starBase v2.0: decoding miRNA-ceRNA, miRNA-ncRNA and protein-RNA interaction networks from large-scale CLIP-Seq data. Nucleic Acids Res. (2014) 42:D92–7. 10.1093/nar/gkt1248 24297251 PMC3964941

[22] Friedman J Hastie T Tibshirani R. Regularization paths for generalized linear models via coordinate descent. J Stat Softw. (2010) 33:1–22. 10.18637/jss.v033.i01 20808728 PMC2929880

[23] Reimand J Isserlin R Voisin V Kucera M Tannus-Lopes C Rostamianfar A. Pathwayenrichment analysis and visualization of omics data using g:Profiler, GSEA, cytoscape and EnrichmentMap. Nat Protoc. (2019) 14:482–517. 10.1038/s41596-018-0103-9 30664679 PMC6607905

[24] Gupta K Miller JD Li JZ Russell MW Charbonneau C. Epidemiologic and socioeconomic burden of metastatic renal cell carcinoma (mRCC): a literature review. Cancer Treat Rev. (2008) 34:193–205. 10.1016/j.ctrv.2007.12.001 18313224

[25] Wang X Yu H Sun W Kong J Zhang L Tang J. The long non-coding RNA CYTOR drives colorectal cancer progression by interacting with NCL and Sam68. Mol Cancer. (2018) 17:110. 10.1186/s12943-018-0860-7 30064438 PMC6069835

[26] Wang WJ Guo CA Li R Xu ZP Yu JP Ye Y. Long non-coding RNA CASC19 is associated with the progression and prognosis of advanced gastric cancer. Aging. (2019) 11:5829–47. 10.18632/aging.102190 31422382 PMC6710062

[27] Yang B Zhang L Cao Y Chen S Cao J Wu D. Overexpression of lncRNA IGFBP4-1 reprograms energy metabolism to promote lung cancer progression. Mol Cancer. (2017) 16:154. 10.1186/s12943-017-0722-8 28946875 PMC5613386

[28] Kong X Duan Y Sang Y Li Y Zhang H Liang Y. LncRNA-CDC6 promotes breast cancer progression and function as ceRNA to target CDC6 by sponging microRNA-215. J Cell Physiol. (2019) 234:9105–17. 10.1002/jcp.27587 30362551

[29] Song J Xu Q Zhang H Yin X Zhu C Zhao K. Five key lncRNAs considered as prognostic targets for predicting pancreatic ductal adenocarcinoma. J Cell Biochem. (2018) 119:4559–69. 10.1002/jcb.26598 29239017 PMC5947154

[30] Lin T Fu Y Zhang X Gu J Ma X Miao R. A seven-long noncoding RNA signature predicts overall survival for patients with early stage non-small cell lung cancer. Aging. (2018) 10:2356–66. 10.18632/aging.101550 30205363 PMC6188476

[31] Liu H Ye T Yang X Lv P Wu X Zhou H. A panel of four-lncRNA signature as a potential biomarker for predicting survival in clear cell renal cell carcinoma. J Cancer. (2020) 11:4274–83. 10.7150/jca.40421 32368310 PMC7196268

[32] Zhang J Zhang X Piao C Bi J Zhang Z Li Z. A long non-coding RNA signature to improve prognostic prediction in clear cell renal cell carcinoma. Biomed Pharmacother. (2019) 118:109079. 10.1016/j.biopha.2019.109079 31351427

[33] Liu H Sun Y Tian H Xiao X Zhang J Wang Y. Characterization of long non-coding RNA and messenger RNA profiles in laryngeal cancer by weighted gene co-expression network analysis. Aging. (2019) 11:10074–99. 10.18632/aging.102419 31739287 PMC6914418

[34] Wang Y Chen L Ju L Qian K Liu X Wang X. Novel biomarkers associated with progression and prognosis of bladder cancer identified by co-expression analysis. Front Oncol. (2019) 9:1030. 10.3389/fonc.2019.01030 31681575 PMC6799077

[35] Fu X Chen G Cai ZD Wang C Liu ZZ Lin ZY. Overexpression of BUB1B contributes to progression of prostate cancer and predicts poor outcome in patients with prostate cancer. Onco Targets Ther. (2016) 9:2211–20. 10.2147/OTT.S101994 27143916 PMC4844448

[36] Wang X Xiao H Wu D Zhang D Zhang Z. miR-335-5p regulates cell cycle and metastasis in lung adenocarcinoma by targeting CCNB2. Onco Targets Ther. (2020) 13:6255–63. 10.2147/OTT.S245136 32636645 PMC7335273

[37] Yan H Zhu C Zhang L. Kinesin family member 18B: a contributor and facilitator in the proliferation and metastasis of cutaneous melanoma. J BiochemMolToxicol. (2019) 33:e22409. 10.1002/jbt.22409 31617652

[38] Fu Z Wen D. The emerging role of polo-like kinase 1 in epithelial-mesenchymal transition and tumor metastasis. Cancers. (2017) 9:131. 10.3390/cancers9100131 28953239 PMC5664070

[39] Song J Ma Q Hu M Qian D Wang B He N. The inhibition of miR-144-3p on cell proliferation and metastasis by targeting TOP2A in HCMV-positive glioblastoma cells. Molecules. (2018) 23:3259. 10.3390/molecules23123259 30544723 PMC6320803

[40] Huang JL Cao SW Ou QS Yang B Zheng SH Tang J. The long non-coding RNA PTTG3P promotes cell growth and metastasis via up-regulating PTTG1 and activating PI3K/AKT signaling in hepatocellular carcinoma. MolCancer. (2018) 17:93. 10.1186/s12943-018-0841-x 29803224 PMC5970477

[41] Jonas S Izaurralde E. Towards a molecular understanding of microRNA-mediated gene silencing. Nat Rev Genet. (2015) 16:421–33. 10.1038/nrg3965 26077373

[42] Yang G Zhang Y Yang J. A five-microRNA signature as prognostic biomarker in colorectal cancer by bioinformatics analysis. Front Oncol. (2019) 9:1207. 10.3389/fonc.2019.01207 31799184 PMC6863365

[43] Zhou X Xu X Wang J Lin J Chen W. Identifying miRNA/mRNA negative regulation pairs in colorectal cancer. Sci Rep. (2015) 5:12995. 10.1038/srep12995 26269151 PMC4534763

[44] Xu YJ Yu H Liu GX. Hsa_circ_0031288/hsa-miR-139-3p/Bcl-6 regulatory feedback circuit influences the invasion and migration of cervical cancer HeLa cells. J Cell Biochem. (2020) 121:4251–60. 10.1002/jcb.29650 32277518

[45] Lou W Ding B Zhong G Du C Fan W Fu P. Dysregulation of pseudogene/lncRNA-hsa-miR-363-3p-SPOCK2 pathway fuels stage progression of ovarian cancer. Aging. (2019) 11:11416–39. 10.18632/aging.102538 31794425 PMC6932902

[46] Chen X Chen Z Yu S Nie F Yan S Ma P. Long noncoding RNA LINC01234 functions as a competing endogenous RNA to regulate cbfb expression by sponging miR-204-5p in gastric cancer. Clin Cancer Res. (2018) 24:2002–14. 10.1158/1078-0432.CCR-17-2376 29386218

[47] Lin C Zhang Y Chen Y Bai Y Zhang Y. Long noncoding RNA LINC01234 promotes serine hydroxymethyltransferase 2 expression and proliferation by competitively binding miR-642a-5p in colon cancer. Cell Death Dis. (2019) 10:137. 10.1038/s41419-019-1352-4 30755591 PMC6372696

[48] Chen Z Chen X Lu B Gu Y Chen Q Lei T. Up-regulated LINC01234 promotes non-small-cell lung cancer cell metastasis by activating VAV3 and repressing BTG2 expression. J HematolOncol. (2020) 13:7. 10.1186/s13045-019-0842-2 31959200 PMC6972004

[49] Zhang C Huang D Liu A Xu Y Na R Xu D. Genome-wide screening and cohorts validation identifying novel lncRNAs as prognostic biomarkers for clear cell renal cell carcinoma. J Cell Biochem. (2020) 121:2559–70. 10.1002/jcb.29478 31646670

[50] Su Y Lu J Chen X Liang C Luo P Qin C. Long non-coding RNA HOTTIP affects renal cell carcinoma progression by regulating autophagy via the PI3K/Akt/Atg13 signaling pathway. J Cancer Res Clin Oncol. (2019) 145:573–88. 10.1007/s00432-018-2808-0 30511250 PMC11810223

[51] Ding J Yeh CR Sun Y Lin C Chou J Ou Z. Estrogen receptor beta promotes renal cell carcinoma progression via regulating LncRNA HOTAIR-miR-138/200c/204/217 associated CeRNA network. Oncogene. (2018) 37:5037–53. 10.1038/s41388-018-0175-6 29789714

[52] Wang J Zhang C He W Gou X. Construction and comprehensive analysis of dysregulated long non-coding RNA-associated competing endogenous RNA network in clear cell renal cell carcinoma. J Cell Biochem. (2018). 10.1002/jcb.27557 30278113

