# Supplementary figures and images for: Construction of Competitive Endogenous RNA Network and Verification of 3-Key LncRNA Signature Associated With Distant Metastasis and Poor Prognosis in Patients With Clear Cell Renal Cell Carcinoma
Source: Front Oncol. 2021 Mar 24;11:640150. doi: 10.3389/fonc.2021.640150 (PMC8044754; doi:10.3389/fonc.2021.640150)

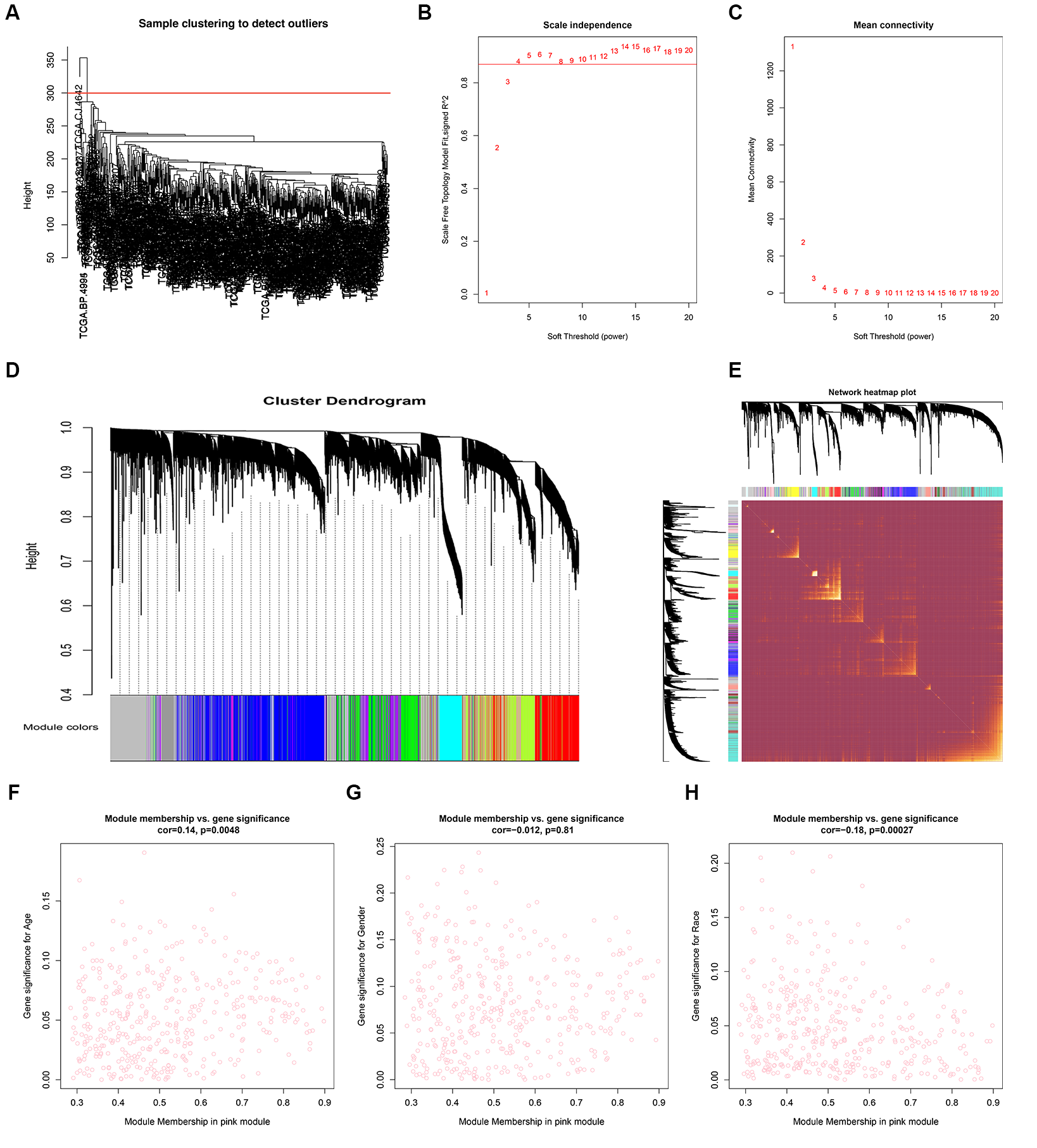

Supplement: Supplementary Figure 1 — Construction of co-expression modules and validation of pink module by WGCNA. (A) The cluster dendrogram of ccRCC samples. Analysis of network topology for different soft-thresholding powers in scale independence (B) and mean connectivity (C). (D) Cluster dendrogram of all DEGs in the TCGA set. Each branch in the dendrogram represents a single gene, and every color below indicates a co-expression gene module. (E) The heatmap describes the TOM among genes based on divided co-expression modules. About 5,000 randomly selected genes were grouped into different modules, which were shown in different color codes below the cluster dendrogram. Light yellow displays a high degree of topological overlap, while dark red indicates a low degree of topological overlap. Scatter plots of GS for age (F), gender (G), and race (H) vs. MM in pink module. The value of p < 0.05 was considered statistically significant. WGCNA, weighted gene co-expression network analysis; ccRCC, Clear cell renal cell carcinoma; TOM, topological overlap matrix; DEGs, differentially expressed genes; GS, gene significance; MM, module membership. [file Image_1.TIF]

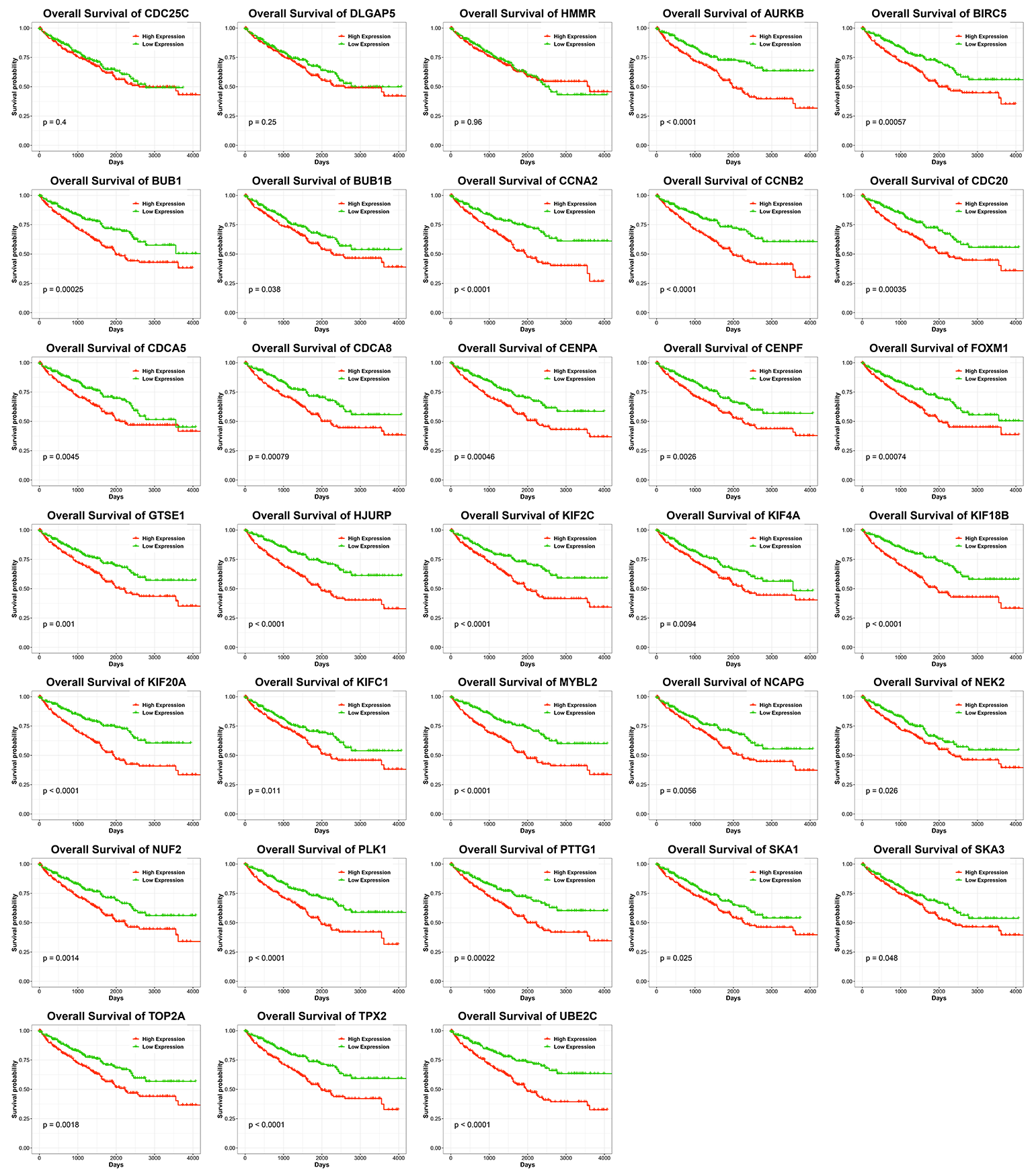

Supplement: Supplementary Figure 2 — Survival analyses of 33 PPI hub mRNAs in the TCGA set. The value of p < 0.05 was considered statistically significant. PPI, protein–protein interaction; TCGA, the cancer genome atlas. [file Image_2.TIF]

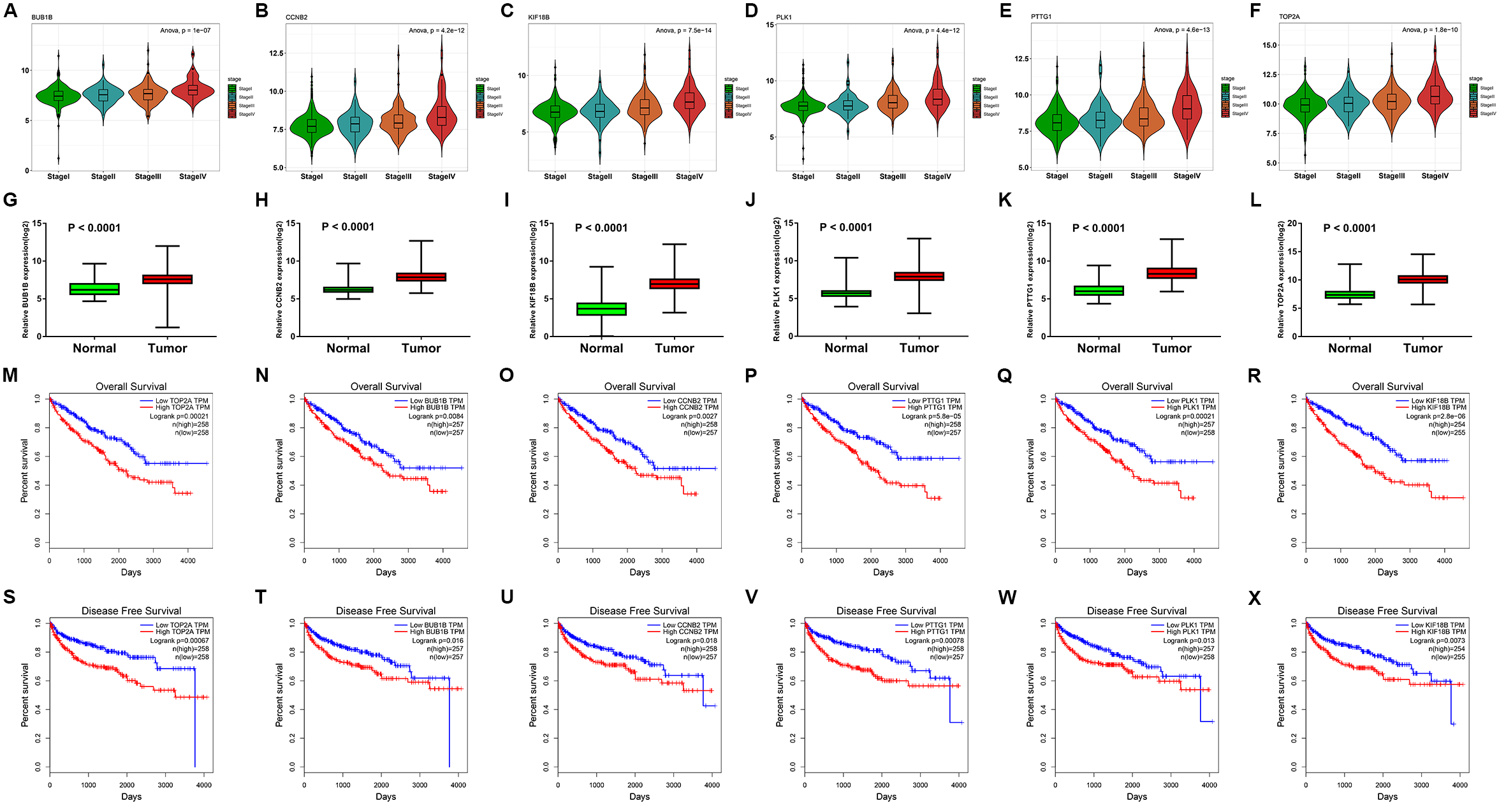

Supplement: Supplementary Figure 3 — Expression analyses and survival validation of six hub mRNAs. Expression analyses of BUB1B (A), CCNB2 (B), KIF18B (C), PLK1 (D), PTTG1 (E), and TOP2A (F) in different stages. Expression analyses of BUB1B (G), CCNB2 (H), KIF18B (I), PLK1 (J), PTTG1 (K), and TOP2A (L) between normal tissues and tumor tissues. OS validation of TOP2A (M), BUB1B (N), CCNB2 (O), PTTG1 (P), PLK1 (Q), and KIF18B (R) in GEPIA database. DFS validation of TOP2A (S), BUB1B (T), CCNB2 (U), PTTG1 (V), PLK1 (W), and KIF18B (X) in GEPIA database. The value of p < 0.05 was considered statistically significant. OS, overall survival; DFS, disease free survival; GEPIA, Gene Expression Profiling Interactive Analysis. [file Image_3.TIF]

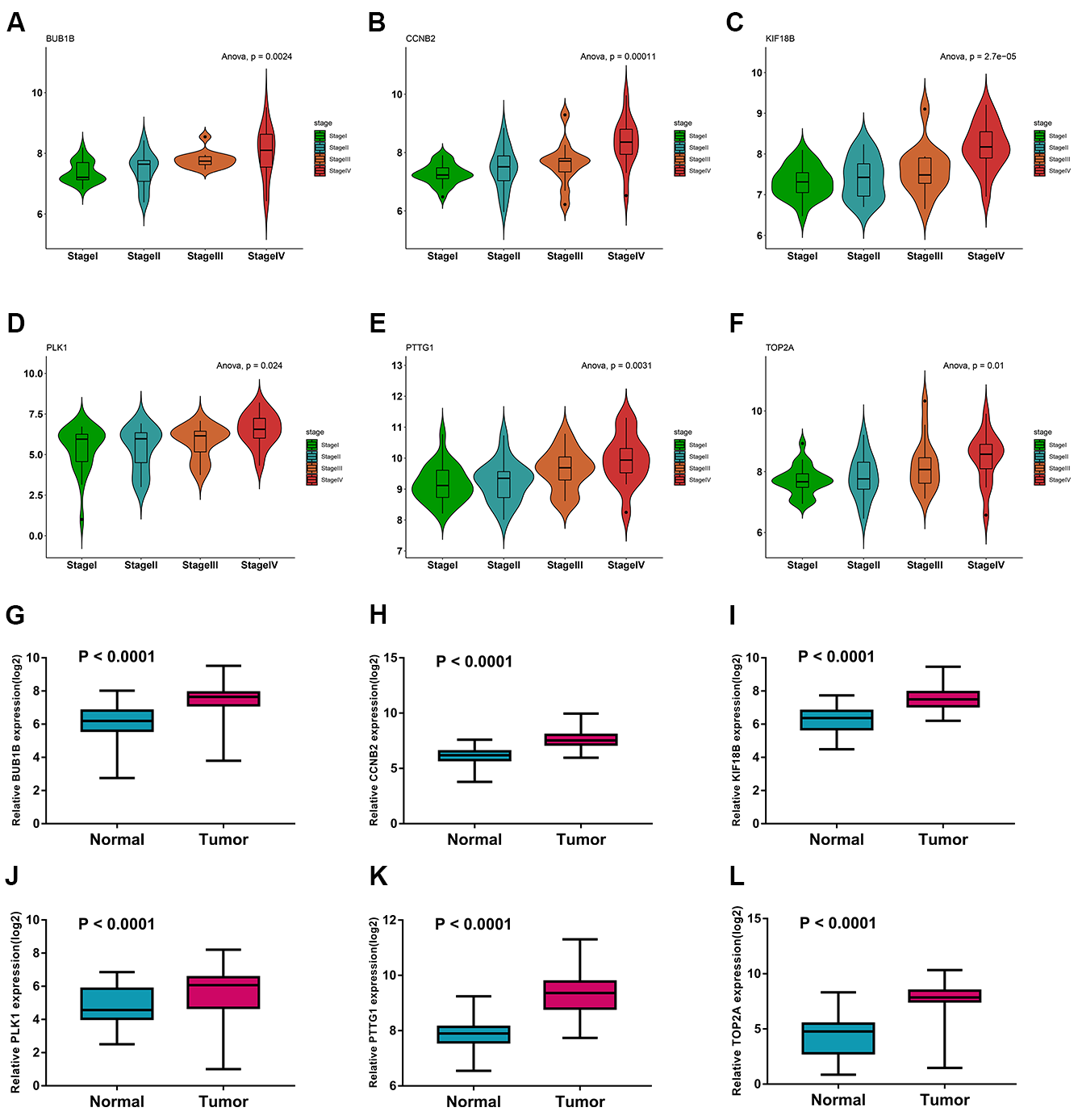

Supplement: Supplementary Figure 4 — Expression pattern validation in GEO dataset. Expression analyses of BUB1B (A), CCNB2 (B), KIF18B (C), PLK1 (D), PTTG1 (E), TOP2A (F) among different stages. Expression levels of BUB1B (G), CCNB2 (H), KIF18B (I), PLK1 (J), PTTG1 (K), TOP2A (L) between tumor tissues and normal tissues. The value of p < 0.05 was considered statistically significant. GEO, Gene expression omnibus. [file Image_4.TIF]

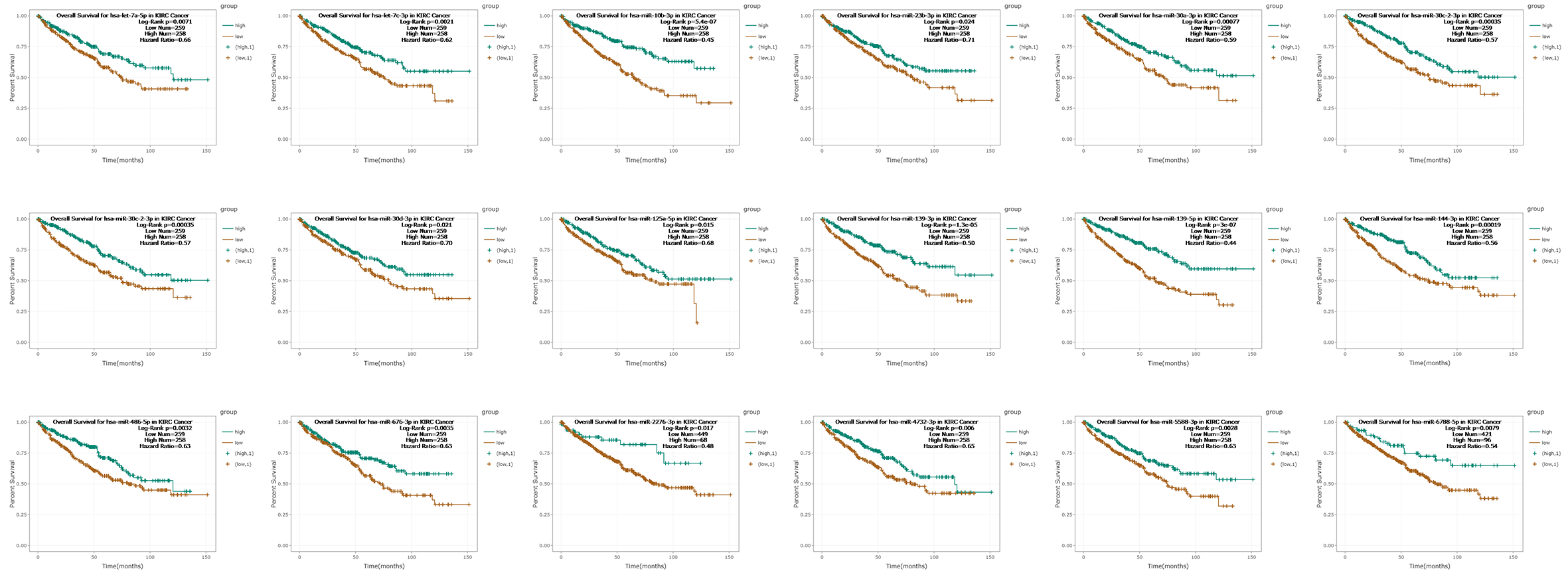

Supplement: Supplementary Figure 5 — Target miRNAs associated with overall survival. The value of p < 0.05 was considered statistically significant. miRNAs, micro RNAs. [file Image_5.TIF]

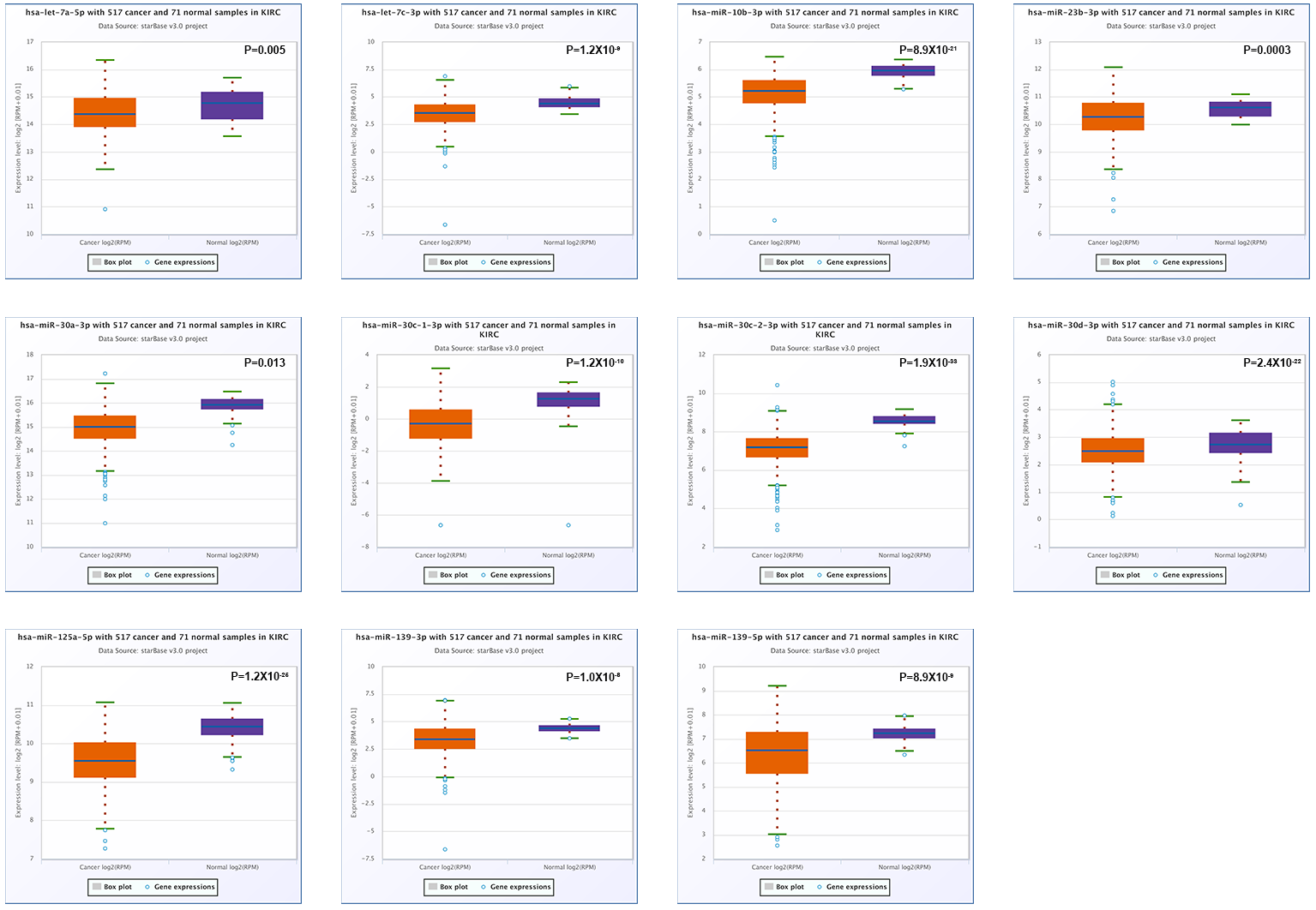

Supplement: Supplementary Figure 6 — Expression levels of 11 tumor suppressor miRNAs between ccRCC/KIRC tissues and normal tissues. The value of p < 0.05 was considered statistically significant. miRNAs, micro RNAs, ccRCC, Clear cell renal cell carcinoma. [file Image_6.TIF]

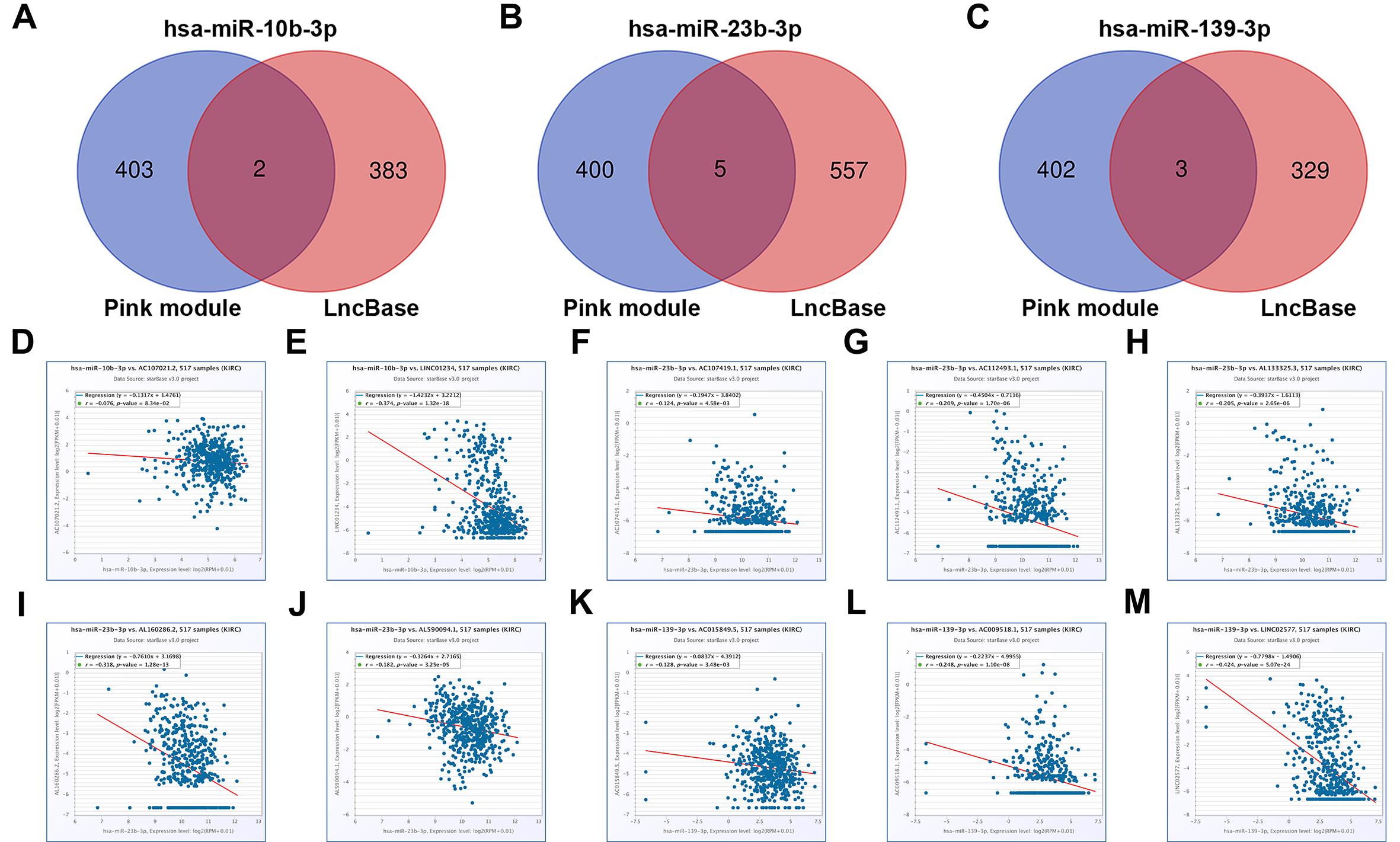

Supplement: Supplementary Figure 7 — Screening of distant metastasis-related lncRNAs by intersection with pink module and expression correlation analyses. Venn diagram of target lncRNAs for hsa-miR-10b-3p (A), hsa-miR-23b-3p (B), and hsa-miR-139-3p (C). (D) The expression correlation between hsa-miR-10b-3p and AC107021.2. (E) The expression correlation between hsa-miR-10b-3p and LINC01234. (F) The expression correlation between hsa-miR-23b-3p and AC107419.1. (G) The expression correlation between hsa-miR-23b-3p and AC112493.1. (H) The expression correlation between hsa-miR-23b-3p and AL133325.3. (I) The expression correlation between hsa-miR-23b-3p and AL160286.2. (J) The expression correlation between hsa-miR-23b-3p and LINC02609/AL590094.1. (K) The expression correlation between hsa-miR-139-3p and AC015849.5. (L) The expression correlation between hsa-miR-139-3p and AC009518.1. (M) The expression correlation between hsa-miR-139-3p and LINC02577. The value of p < 0.05 was considered statistically significant. lncRNAs, Long non-coding RNAs. [file Image_7.TIF]
